# Interrelationships between Yeast Ribosomal Protein Assembly Events and Transient Ribosome Biogenesis Factors Interactions in Early Pre-Ribosomes

**DOI:** 10.1371/journal.pone.0032552

**Published:** 2012-03-14

**Authors:** Steffen Jakob, Uli Ohmayer, Andreas Neueder, Thomas Hierlmeier, Jorge Perez-Fernandez, Eduard Hochmuth, Rainer Deutzmann, Joachim Griesenbeck, Herbert Tschochner, Philipp Milkereit

**Affiliations:** 1 Lehrstuhl für Biochemie III, Universität Regensburg, Regensburg, Germany; 2 Lehrstuhl für Biochemie I, Universität Regensburg, Regensburg, Germany; University of Edinburgh, United Kingdom

## Abstract

Early steps of eukaryotic ribosome biogenesis require a large set of ribosome biogenesis factors which transiently interact with nascent rRNA precursors (pre-rRNA). Most likely, concomitant with that initial contacts between ribosomal proteins (r-proteins) and ribosome precursors (pre-ribosomes) are established which are converted into robust interactions between pre-rRNA and r-proteins during the course of ribosome maturation. Here we analysed the interrelationship between r-protein assembly events and the transient interactions of ribosome biogenesis factors with early pre-ribosomal intermediates termed 90S pre-ribosomes or small ribosomal subunit (SSU) processome in yeast cells. We observed that components of the SSU processome UTP-A and UTP-B sub-modules were recruited to early pre-ribosomes independently of all tested r-proteins. On the other hand, groups of SSU processome components were identified whose association with early pre-ribosomes was affected by specific r-protein assembly events in the head-platform interface of the SSU. One of these components, Noc4p, appeared to be itself required for robust incorporation of r-proteins into the SSU head domain. Altogether, the data reveal an emerging network of specific interrelationships between local r-protein assembly events and the functional interactions of SSU processome components with early pre-ribosomes. They point towards some of these components being transient primary pre-rRNA *in vivo* binders and towards a role for others in coordinating the assembly of major SSU domains.

## Introduction

**Figure 1:**
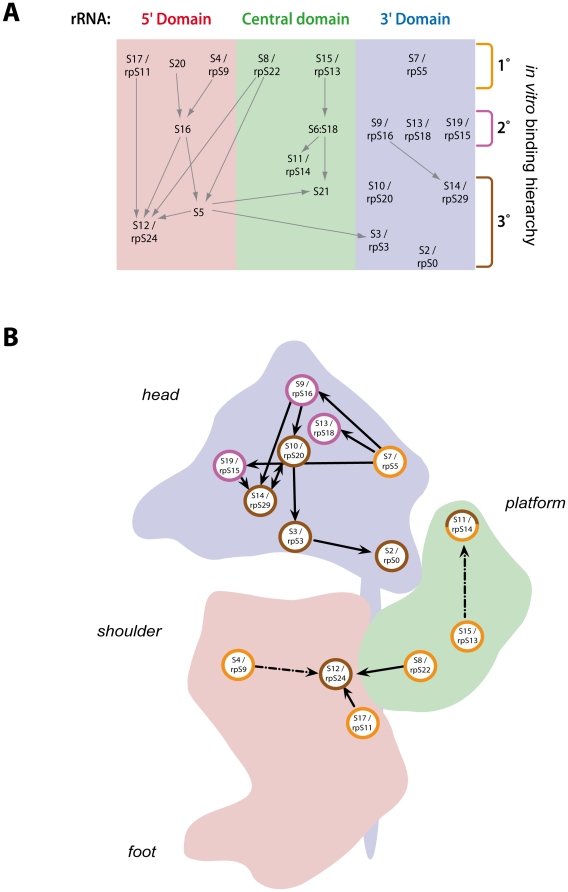
30S *in vitro* assembly map ordered in accordance to the domain organisation of the 16S rRNA and represented in a 2D projection of the 30S ribosomal subunit (adapted from [**4**]). (**A**) The six different r-protein assembly trees (initiated by primary binding r-proteins) of the *E. coli* 30S subunit are ordered according to their physical location on the 16S rRNA (in 5′ to 3′ direction) and attributed to 16S rRNA domain organisation (5′, central, and 3′ domain). The r-proteins are classified by their binding hierarchy. Primary binding proteins (1°) are capable of initiating pioneering interactions with rRNA independent of other proteins. The secondary binders (2°) require one or more primary binding proteins for their association with rRNA, while tertiary binding (3°) proteins require both primary and secondary binders for their incorporation into ribosomal subunits. If existing, homologous r-proteins in *S. cerevisiae* (rpS nomenclature) are shown next to their prokaryotic counterparts. (**B**) A schematic presentation of the tertiary structure of the 16S rRNA is depicted. Each of the three major secondary structure domains of the 16S rRNA forms distinct morphological features of the 30S subunit. The 16S rRNA 5′ domain forms the shoulder and foot (red), the central domain forms the platform (green) and the 3′ major domain forms the head (blue). The assembly map of (A) is superimposed in this schematic structure visualisation paying attention to the localisation of the respective r-protein. The colour of the circle gives information about the assembly hierarchy of the respective r-protein (see also A). S11/rpS14 is classified in a species-dependent manner as a tertiary binder (*E. coli*) or a primary binder (*Aquifex aeolicus*) [60]. Only r-proteins with sequence homologous in *S. cerevisiae* (rpS nomenclature) are shown. The figure is reproduced and adapted from [4] (adaptation from the original assembly map of Nomura and colleagues [1]).

Prokaryotic ribosomes consist of three ribosomal RNAs (rRNAs) and ∼55 ribosomal proteins (r-proteins). *In vitro* assembly of prokaryotic ribosomes may occur in the absence of auxiliary factors and follows hierarchical principles [1]–[4]. Primary binding r-proteins are capable of initiating interactions with the rRNA independently of other proteins. Secondary binders require one or more primary binding proteins for their stable association with rRNA, while tertiary binding proteins require both primary and secondary binders for their efficient incorporation into ribosomal subunits. According to the primary binding event, r-proteins of the small ribosomal subunit (SSU) can be grouped into six different assembly trees, each of which assembles in a cooperative manner. R-proteins of three of these assembly trees bind to the 5′ secondary structure domain of the prokaryotic 16S SSU rRNA, r-proteins of two other assembly trees bind to the central domain, and r-proteins of the sixth assembly tree bind to the 3′ major domain (see Fig. 1). Each of the three major secondary structure domains of the 16S rRNA forms distinct morphological features of the SSU: the 5′ domain forms the shoulder and the foot, the central domain forms the platform and the 3′ major domain forms the head. Remarkably, these three major SSU rRNA domains can largely assemble *in vitro* with corresponding r-proteins independently of each other [5]–[7]. More recently, time resolved hydroxyl radical footprinting analyses showed that some of the contacts of r-proteins with the 16S rRNA can already be observed very soon after initiating prokaryotic SSU *in vitro* assembly reactions [8]. The establishment of other contacts, however, was substantially slower, probably driven by induced fit mechanisms.

Eukaryotic ribosomes consist of four rRNAs and ∼80 r-proteins. Studies in the yeast *S. cerevisiae* indicate that both the gradual establishment of high affinity interactions between r-proteins and rRNA and the hierarchy of individual r-protein-rRNA assembly events also apply to the *in vivo* formation of eukaryotic ribosomes [9]–[11]. On the other hand, around 150 non-ribosomal factors have been described to be essential for ribosome biogenesis in yeast [12], with many of them thought to facilitate ribosome assembly. A substantial number of these factors are required for early steps of yeast SSU maturation. These proteins are part of an early pre-ribosomal particle with an estimated sedimentation coefficient of approximately 90S which contains furthermore the 35S rRNA precursor and the U3 small nucleolar (sno) RNA [13]–[16]. The particle was referred to as 90S pre-ribosome [16] or the SSU processome [15] and many of its non-ribosomal protein components were named U three proteins (Utp).

Several protein sub-complexes of the SSU processome could be purified as separate entities from yeast cell extracts depleted of pre-ribosomal particles by a high speed centrifugation step [17]. Amongst them is the UTP-A/t-UTP subcomplex [17], [18] (Utp4p, Utp8p, Utp9p, Utp10p, Utp15p, Nan1p, Utp5p and Pol5p), the UTP-B/Pwp2p subcomplex [17], [19] (Pwp2p, Dip2p, Utp6p, Utp13, Utp18p, and Utp21p), the UTP-C subcomplex [17] (Utp22p, Rrp7p, Cka1p, Cka2p, Ckb1p, and Ckb2p), a sub-module containing Rcl1p and Bms1p [17], [20], and a ribonucleoprotein complex containing besides U3 snoRNA and Rrp9p the proteins Nop1p, Nop56p and Nop58p [17], [21], [22]. Other subcomplexes of the SSU processome could be reconstituted *in vitro* from recombinant components, as the human MPP10 complex, consisting of the human counterparts of yeast Mpp10p, Imp3p, and Imp4p [23], and a complex consisting of yeast Noc4p and Nop14p [24], [25]. Several of these SSU processome subcomplexes were shown to associate in a hierarchical order with rRNA precursors [26]–[28]. Both SSU processome components and, at least some r-proteins are thought to associate *in vivo* with nascent rRNA precursors already during transcription of the precursor rRNA gene [11], [15], [29], [30].

In this study, we aimed to analyse the relationship between individual r-protein assembly events and the association of SSU processome components with rRNA precursors. Beside the possibility that eukaryotic SSU processome components might trigger assembly of specific r-proteins with rRNA, a few major scenarios are conceivable whether and how r-protein assembly events could affect the transient SSU processome association with rRNA precursors. (I) SSU processome components initiate rRNA contact and associate independent of r-protein(s). (II) SSU processome components and r-protein(s) associate cooperatively with rRNA precursors. (III) The association of SSU processome components requires the preceding binding of r-protein(s). (IV) R-protein assembly might trigger release of SSU processome components from rRNA precursors.

To distinguish between these possibilities, several yeast conditional mutant strains have been established in this work allowing to analyse the association of SSU processome subcomplexes with early pre-ribosomes depleted of representative r-proteins of each structural domain of the 18S rRNA (5′, central and 3′ domain). In summary, the results of these analyses indicated a network of specific interrelationships between local r-protein assembly events and the functional interactions of SSU processomal submodules with early pre-ribosomes.

## Results

### Analysis of UTP-A and UTP-B association with early pre-ribosomes in yeast strains *in vivo* depleted of SSU r-proteins

**Figure 2:**
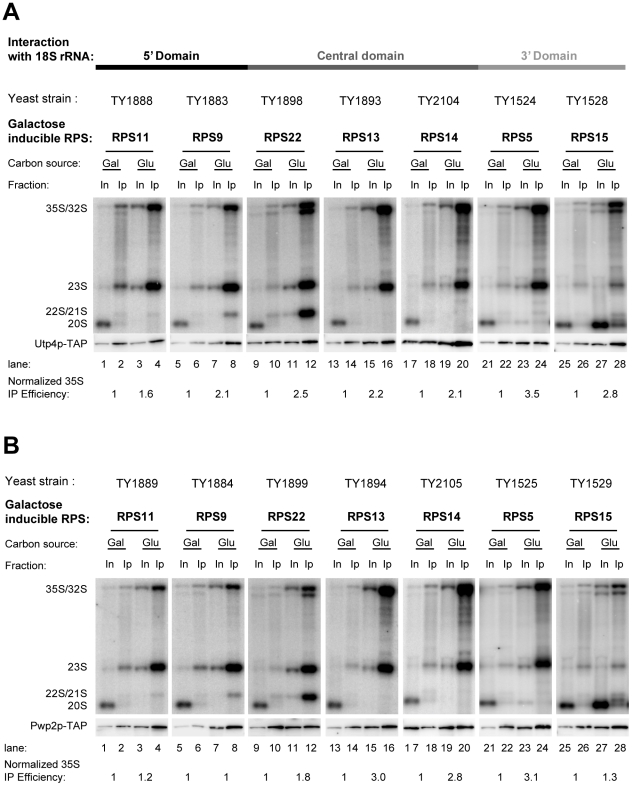
Analysis of pre-rRNAs co-purifying with UTP-A or UTP-B components after *in vivo* depletion of r-proteins of the SSU. The indicated yeast strains carrying galactose inducible alleles of the indicated SSU r-protein genes in combination with TAP-tag fusion alleles of UTP-A component Utp4p (**A**), or UTP-B component Pwp2p (**B**), were either cultivated in medium containing galactose (Gal) as carbon source or were transferred to glucose containing medium (Glu) and cultivated for additional four hours to turn off the expression of the respective r-proteins. TAP-tagged bait proteins were affinity purified via their Protein A moiety using IgG sepharose beads. The amount of purified bait protein was monitored by Western blotting (lower panels) and co-purified pre-rRNA species were analysed by Northern blotting (upper panels) using oligo 1819, which hybridizes in ribosomal precursor rRNAs between 18S and 5.8S rRNA sequences and detects 35S, 32S, 23S, and 20S pre-rRNAs (see Fig. S1). Equal signal intensities of input (In) and beads (IP) fractions in Northern blots correspond to 1% co-precipitation of the respective rRNA. Efficiencies of 35S pre-rRNA purification normalized to the values obtained for cells grown in permissive conditions are indicated in the lower panels. For the Western blot analyses equal signal intensities of input (In) and beads (IP) correspond to 20% precipitation of the TAP-tagged bait protein. The strains are ordered in regard to the binding of the respective r-proteins to the three major secondary structure domains of the 18S rRNA. Prokaryotic homologues of rpS11, rpS9, rpS22, rpS13, and rpS5 are primary rRNA *in vitro* binders. Prokaryotic homologues of rpS15 and rpS14 are secondary/tertiary *in vitro* binders of the assembly trees initiated by binding of the homologues of rpS13 and rpS5, respectively (see Fig. 1).

To analyse possible hierarchical relationships between recruitment of SSU processome sub-modules to yeast pre-rRNA and r-protein assembly events we constructed a set of yeast conditional r-protein gene mutants expressing epitope tagged variants of SSU processome components. First, we wanted to test how pre-ribosome association of the UTP-A member Utp4p and the UTP-B member Pwp2p is affected in strains depleted of rpS11, rpS9, rpS22, rpS13, and rpS5 (yeast homologues of five *E. coli* primary *in vitro* binders) or in strains depleted of rpS15 and rpS14 (yeast homologues of *E. coli* secondary and tertiary *in vitro* binder, respectively) which bind to different regions of the SSU rRNA ([31], [32], see also Fig. 1). Yeast conditional mutant strains expressing the above mentioned ribosomal protein genes under the control of a galactose inducible promoter [9] were modified by tagging chromosome encoded Utp4p or Pwp2p with the tandem affinity purification (TAP) tag [33]. Expression shut down of the selected rpS by shifting the corresponding yeast mutant strains for four hours to glucose containing medium prevents their assembly into newly synthesized ribosomal particles and leads to specific pre-rRNA processing phenotypes [9]. Accordingly, depletion of rpS9 and rpS11, the homologous of the *E. coli* primary *in vitro* binders of the 18S rRNA 5′ domain, and depletion of rpS13 and rpS14, homologous of the *E. coli* - primary and tertiary binders of the central domain, led to a strong accumulation of 35S and 23S pre-rRNAs, while 20S pre-rRNA was not any more detectable (Fig. 2A–B, compare 32/35S signals in lanes 1,5,13, and 17 with 32/35S signals in lanes 3,7,15 and 19, respectively, see also Fig. S1 for a scheme of yeast rRNA processing and Fig. 1 for an illustration of the *in vitro* assembly map of the *E. coli* SSU). Such a rRNA processing phenotype is consistent with a strong delay of early SSU processome dependent processing events in the 5′ external transcribed spacer (5′-ETS) at A_0_ and A_1_ and in the internal transcribed spacer 1 (ITS-1) at site A_2_. Depletion of rpS22, the homologue of the second *E. coli* primary *in vitro* binder of the central domain, resulted also in accumulation of 35S and 23S pre-rRNAs. In addition, a pre-rRNA species migrating slightly faster than 23S pre-rRNA accumulated in this strain, indicating residual processing at site A_0_ (Fig. 2A–B, compare lane 11 with lane 9). Shut down of expression of rpS5, the homologue of the primary *E. coli in vitro* binder of the 3′ major domain, led to some residual appearance of 20S pre-rRNA, indicating that processing in the 5′-ETS and ITS-1 at sites A_0_, A_1_, and A_2_ was strongly affected, but not completely blocked in this strain (Fig. 2A–B, compare lane 23 with lane 21). In contrast, accumulation of 20S pre-rRNA in strains depleted of rpS15, homologue of the *E. coli in vitro* secondary binder of the 3′ domain, showed that processing in the 5′-ETS at sites A_0_ and A_1_ and in the ITS-1 at site A_2_ could still efficiently occur (Fig. 2A–B, compare lane 27 with lane 25). These observed pre-rRNA processing phenotypes were in good agreement with the ones previously observed after knock downs of yeast [9] and human [34] ribosomal protein genes. In several cases (RPS11, RPS9, RPS13, RPS14, RPS5) they resembled the ones seen in yeast strains mutated in genes of SSU processome components [15].

When Utp4p-TAP and Pwp2p-TAP were affinity purified from extracts of the corresponding yeast strains grown at permissive conditions efficient co-purification of 23S, 32S, and 35S pre-rRNAs was observed, indicative for their expected association with early pre-ribosomes (Fig. 2A–B, compare lanes 1,5,9,13,17,21 and 25 with lanes 2,6,10,14,18,22 and 26). *In vivo* depletion of none of the tested r-proteins led to a significant reduction in association of Utp4p-TAP or Pwp2p-TAP with early 32/35S pre-rRNA containing pre-ribosomes (Fig. 2A–B, compare 32/35S signals in lanes 3, 7, 11, 15, 19, 23 and 27 with 32/35S signals in lanes 4, 8, 12, 16, 20, 24 and 28 respectively). In most of the cases 32/35S pre-rRNAs co-purified with higher efficiency (up to 3.5 fold increase in purification efficiency) with these SSU processome components, suggesting that their interaction with pre-ribosomal particles was even stabilized. Moreover, Utp4p-TAP and Pwp2p-TAP stayed associated with partially processed 23S and 22S/21S pre-rRNA accumulating in the analyzed ribosomal protein gene mutants.

In summary, these results showed that none of the analysed r-protein assembly events are important for efficient association of members of the UTP-A and UTP-B SSU processome sub-modules with early pre-ribosomes. The data furthermore indicated that their average dwell time on pre-ribosomes increases in the absence of the tested r-proteins.

### Analysis of the protein composition of early pre-ribosomes in yeast mutants affected in 18S rRNA 3′ or central domain assembly events

**Figure 3:**
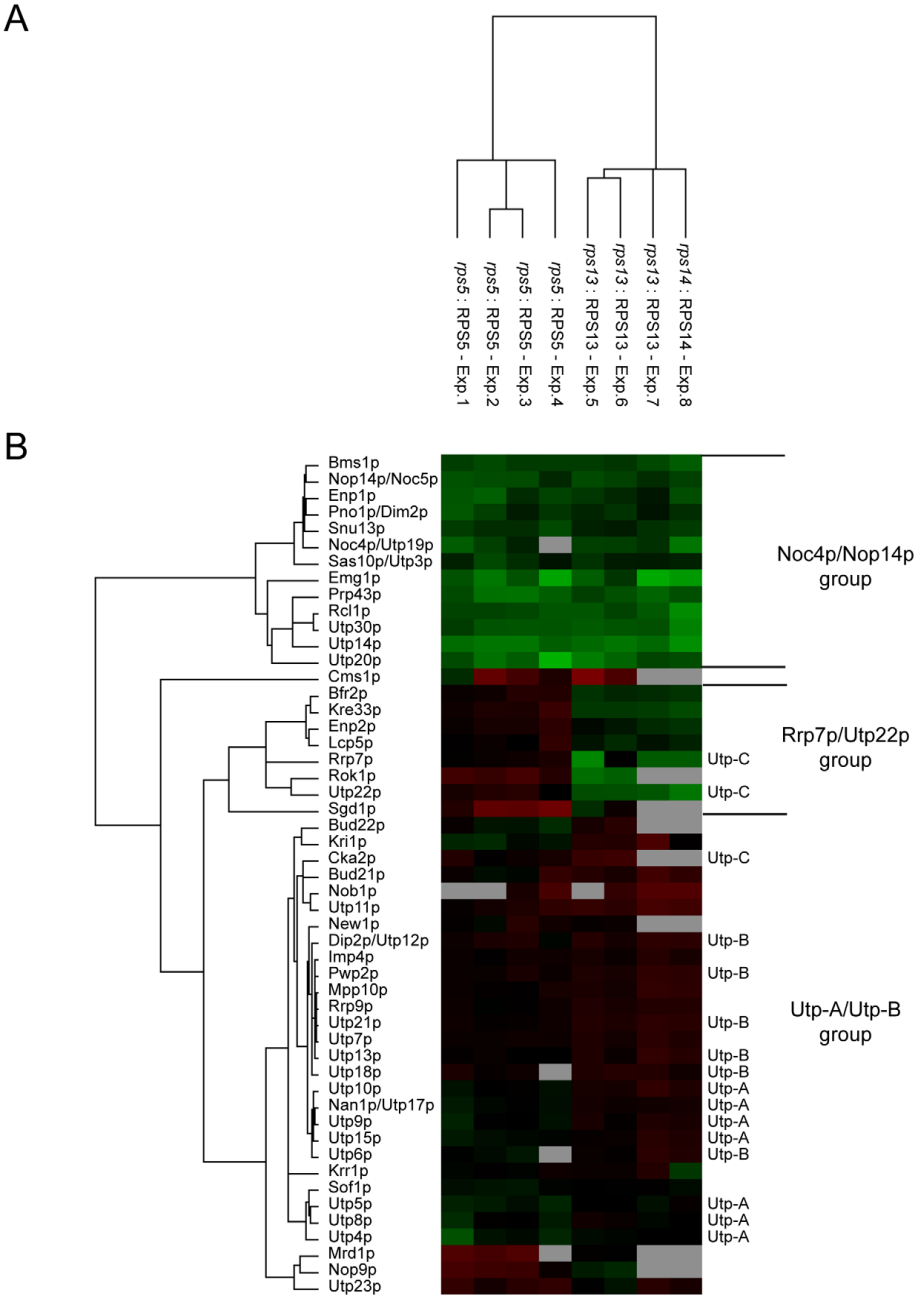
Analysis of changes in ribosome biogenesis factor composition of early 40S pre-ribosomes purified from cells after *in vivo* depletion of SSU r-proteins rpS5, rpS13, or rpS14. The yeast strain TY1907 (wildtype) expressing chromosome encoded TAP tagged Utp4p, and conditional mutant yeast strains expressing chromosome encoded TAP tagged Utp4p and carrying in addition galactose inducible alleles of RPS5 (TY1524), RPS13 (TY1893), or RPS14 (TY2104) were cultivated in medium containing galactose as carbon source and were subsequently transferred to glucose containing medium and cultivated for additional four hours. Utp4p-TAP was affinity purified from corresponding cellular extracts using IgG coupled magnetic beads. Affinity purified proteins were digested by trypsin and the resulting peptides from each sample were labelled with specific iTRAQ reagents. Labelled peptides of wildtype samples were combined with labelled peptides of samples derived from the conditional mutants of either RPS5, RPS13, or RPS14 and were then further analyzed by LC-MS/MS as described in material and methods. Datasets of in total eight (mutant:wildtype) comparisons were generated. In experiments 1–4 Utp4p-TAP fractions purified from the wildtype strain (TY1907) were compared with Utp4p-TAP fractions purified from the conditional RPS5 mutant (TY1524). In experiments 5–7 Utp4p-TAP fractions purified from the wildtype strain (TY1907) were compared with the ones purified from the conditional RPS13 mutant strain (TY1893). In experiment 8 Utp4p-TAP fractions from the wildtype strain were compared with the one purified from the conditional RPS14 mutant strain (TY2104). Experiments 3 and 6 are duplicates of the LC-MS/MS analysis of experiments 2 and 5, respectively. iTRAQ ratios of SSU processome components identified in 5 or more of the 8 experiments were combined to one dataset and statistical clustering algorithms were applied as described in material and methods. (**A**) shows a comparison of the similarity of the eight individual experimental datasets in regard to each other and (**B**) shows a clustering analysis of the identified SSU processome components in regard to their iTRAQ ratios in the eight experiments. In (B) boxes in red colours represent relative enrichment and boxes in green colours relative deprivation of a protein in Utp4p-TAP fractions purified from mutant versus wildtype cells. Boxes in gray colour indicate that no peptide of the respective protein could be identified in the corresponding experiment. Standard names of the identified components are indicated in (B) on the left. On the right the major protein groups described in the text are designated and it is indicated if a component belongs to the UTP-A, UTP-B, or UTP-C SSU processome sub-module. We note that despite the overall highly similar composition of Utp4-TAP purifications from cells *in vivo* depleted of rps13 (experiments 5–7) or rpS14 (experiment 8) the dataset gave first indications for specific differences between these pre-ribosomal populations, as in the content of Krr1p.

To analyse the role of individual r-proteins in SSU processome sub-module association with early pre-ribosomes on a more global level we studied the ribosome biogenesis factor composition of early ribosomal precursor complexes produced in yeast conditional r-protein gene mutants. Pre-ribosomes were affinity purified from yeast conditional mutant strains in which expression of the 3′ domain constituent rpS5 or the central domain constituents rpS13 or rpS14 was shut down. RpS5 and rpS14 are located adjacent to each other in the cleft formed between the head and the platform of the SSU [31], [32]. Association of S11, the prokaryotic homologue of rpS14, with rRNA depends *in vitro* on previous assembly of S15, the prokaryotic homologue of rpS13 (see Fig. 1). According to the results shown in Figure 2, association of Utp4p-TAP with early pre-ribosomes is not reduced in any of the corresponding conditional r-protein gene mutants (Figure 2A, compare input lanes with Ip lanes in glucose conditions). Utp4p-TAP was affinity purified from cultures of wildtype cells and from cultures of the respective conditional r-protein gene mutants shifted to restrictive conditions. Affinity purified Utp4p-TAP fractions were analyzed by semi-quantitative mass spectrometry as indicated in Materials and Methods [35], [36]. The experiments were repeated several times and a dataset of in total eight comparisons between Utp4p-TAP fractions purified from wildtype cells with the ones purified from conditional yeast mutants of RPS5, RPS13 or RPS14 was further analysed by statistical clustering algorithms. More than 50 SSU processome components, identified in total by 249 to 485 peptides in the individual experiments (confidence interval >95% for individual peptides), could be detected in five or more of the eight experiments and were included in the statistical analysis. The statistical analysis indicated that the ribosome biogenesis factor composition of early pre-ribosomes prepared from rpS13 and rpS14 depleted cells were largely similar to each other but differed from the ones purified from rpS5 depleted cells and from wildtype cells (see Fig. 3A). This observation argued for the experimental setup being sufficiently robust for a comparison of the ribosome biogenesis factor composition of early pre-ribosomal particles. As shown in Figure 3B the analyses revealed three main groups of SSU processome components (Noc4p/Nop14p group, Utp22p/Rrp7p group and UTP-A/UTP-B group). Individual members of one group behaved similar to each other but significantly differed in their co-purification with Utp4p-TAP when compared to members of the other groups. In contrast to most members of the UTP-A/UTP-B group, members of the Noc4p/Nop14p group tended to be underrepresented in pre-ribosomes depleted of either rpS5, rpS13, or rpS14. Members of the third major group, the Utp22p/Rrp7p group, were by tendency underrepresented in pre-ribosomes depleted of central domain binders rpS13 and rpS14, but not after depletion of the primary head domain binder rpS5.

In summary, these analyses suggested that stable binding of two defined, overlapping groups of SSU processome components to early pre-ribosomes is affected by inhibition of specific 18S rRNA central or 3′ domain assembly events.

### Detailed analysis of the impact of specific r-protein assembly events on the association of Noc4p with early pre-ribosomes

**Figure 4:**
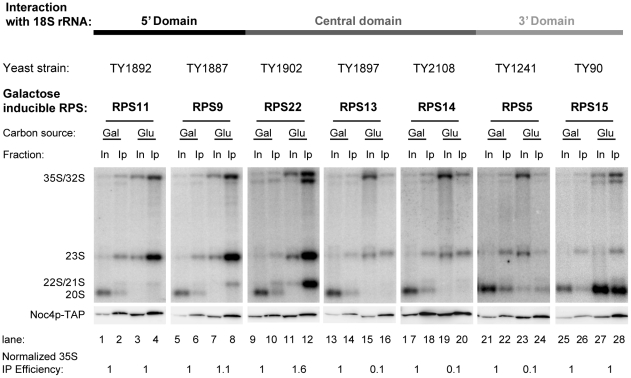
Analysis pre-rRNAs co-purifying with Noc4p-TAP after *in vivo* depletion of r-proteins of the SSU. The yeast strains carrying galactose inducible alleles of the indicated SSU r-protein genes in combination with TAP-tag fusion alleles of Noc4p were either cultivated in medium containing galactose (Gal) as carbon source or were transferred to glucose containing medium (Glu) and cultivated for additional four hours. Noc4p-TAP was affinity purified via its Protein A moiety using IgG sepharose beads. The amount of purified Noc4p-TAP was monitored by Western blotting (lower panels) and co-purified pre-rRNA species were analysed by Northern blotting (upper panels) using oligo 1819, which hybridizes in ribosomal precursor rRNAs between 18S and 5.8S rRNA sequences and detects 35S, 32S, 23S, and 20S pre-rRNAs (see Fig. S1). Equal signal intensities of input (In) and beads (IP) fractions in Northern blots correspond to 1% co-precipitation of the respective rRNA. Efficiencies of 35S pre-rRNA purification normalized to the values obtained for cells grown in permissive conditions are indicated in the lower panel. For the Western blot analyses equal signal intensities of input (In) and beads (IP) correspond to 20% precipitation of the TAP-tagged bait protein. The strains are ordered in regard to the binding of the respective r-proteins to the three main secondary structure domains of the 18S rRNA. Prokaryotic homologues of rpS11, rpS9, rpS22, rpS13, and rpS5 are primary rRNA *in vitro* binders. Homologues of rpS15 and rpS14 are secondary/tertiary *in vitro* binders in the assembly trees initiated by binding of the homologues of rpS13 and rpS5, respectively (see Fig. 1).

**Figure 5:**
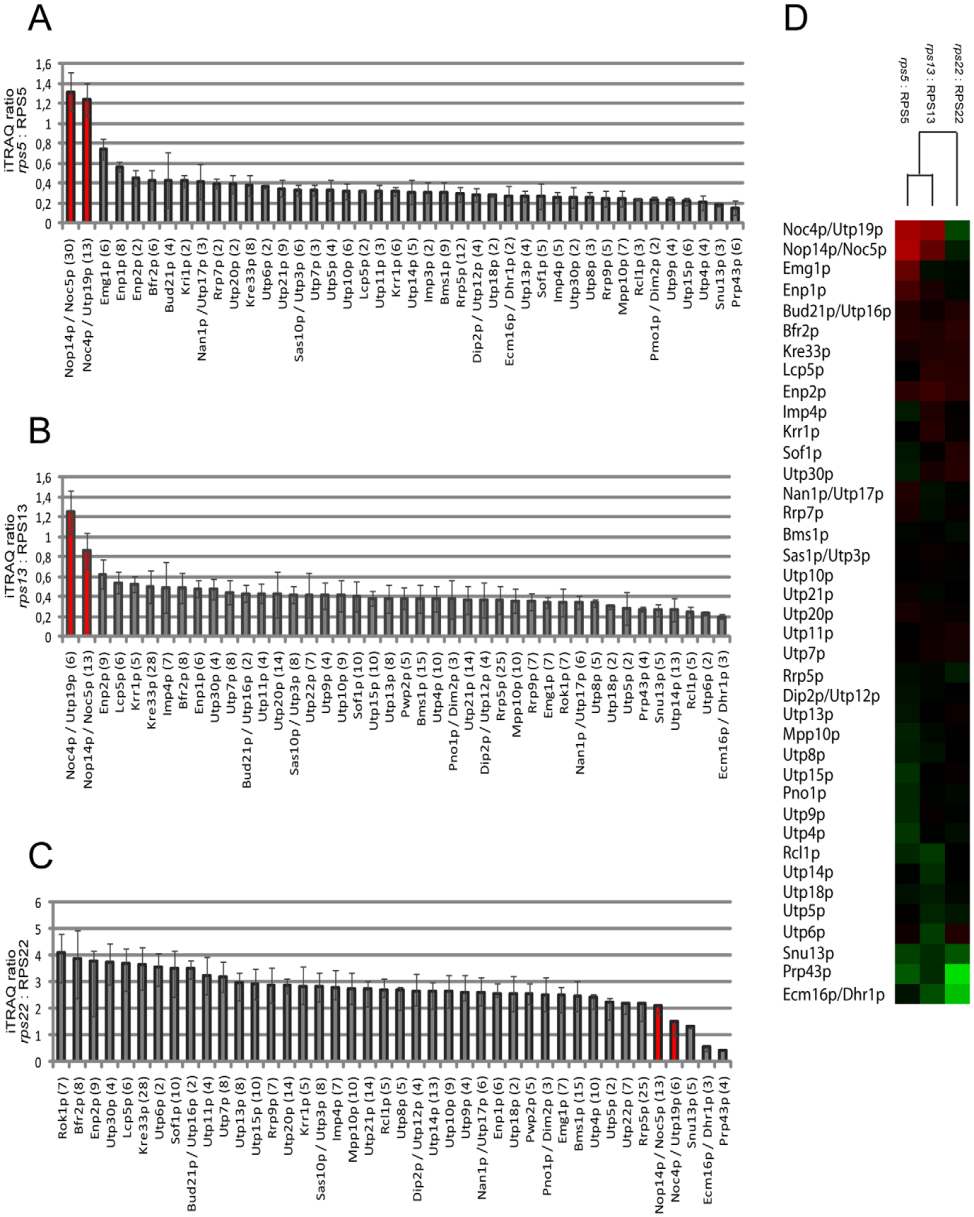
Analysis of ribosome biogenesis factors co-purifying with Noc4p after *in vivo* depletion of rpS5, rpS13, or rpS22. The yeast strain TY96 (wildtype) expressing chromosome encoded TAP tagged Noc4p and yeast conditional mutant strains TY1241, TY1897, and TY1902, expressing chromosomal encoded TAP tagged Noc4p and carrying in addition galactose inducible conditional alleles of RPS5 (TY1241), RPS13 (TY1897), or RPS22 (TY1902) were cultivated in medium containing galactose as carbon source and were subsequently transferred to glucose containing medium and cultivated for additional four hours. Noc4p-TAP was affinity purified from corresponding cellular extracts using IgG coupled magnetic bead matrix. Affinity purified proteins were digested using trypsin and the resulting peptides from each sample were labelled with different iTRAQ reagents. Labelled peptides of wildtype samples were combined with labelled peptides of samples derived either from the conditional mutant of RPS5 (**A**), RPS13 (**B**), or RPS22 (**C**) and were then further analyzed as described in material and methods. Average iTRAQ ratios of each SSU processome component identified by more than one peptide are indicated in (A)–(C). Numbers in brackets behind SSU processome component names indicate the number of peptides (confidence interval >95%) by which the respective protein was identified. (**D**) shows a heatmap representation of the three datasets. The factors are ordered according to a clustering analysis (see material and methods). Boxes in red colours represent relative enrichment and boxes in green colours relative deprivation of a protein in Noc4p-TAP fractions purified from mutant versus wildtype cells.

Noc4p was identified above as a member of the Noc4p/Nop14p group of SSU processome components tending to be underrepresented in early pre-ribosomes purified from yeast cells depleted of rpS5, rpS13, or rpS14. We were interested to characterize in more detail the influence of r-protein assembly events on association of Noc4p with early pre-ribosomes. Conditional mutants of several SSU r-protein genes were constructed which express a chromosome encoded C-terminal TAP-fusion allele of Noc4p. The selected conditional r-protein gene mutants were the same as the ones studied in the experiments shown in Figure 2 and therefore included again the head domain binder rpS15 and the central domain/platform binder rpS14 together with the yeast homologues of five primary *E. coli in vitro* binders interacting with different regions of the SSU rRNA (see Fig. 1). Noc4p-TAP was affinity purified from extracts of these mutants either grown in permissive or restrictive conditions. As expected for a SSU processome component, Northern blot analyses indicated that Noc4p-TAP co-purified significant amounts of early SSU rRNA precursors (23S and 32/35S pre-rRNAs) from extracts of cells grown in permissive conditions (Fig. 4, compare 23S and 32/35S signals in lanes 1, 5, 9, 13, 17, 21 and 25 with 32/35S signals in lanes 2, 6, 10, 14, 18, 22 and 26). *In vivo* depletion of the various r-proteins led to the expected pre-rRNA processing phenotypes (Fig. 4, compare 32/35S signals in lanes 1, 5, 9, 13, 17, 21 and 25 with 32/35S signals in lanes 3,7,11,15,19,23 and 27, compare also with Fig. 2 and [9]). Interestingly, Noc4p-TAP efficiently co-purified large amounts of accumulating early 32/35S pre-rRNAs from extracts of a subset of the analyzed r-protein gene mutants shifted to restrictive conditions (RPS11, RPS9, RPS22, RPS15, see Fig. 4, compare 32/35S signals in lanes 3, 7, 11 and 15 with signals in lanes 4, 8, 12 and 16). By contrast, the efficiency of co-purification of early 32/35S pre-rRNA with Noc4p-TAP from extracts of strains depleted of another subset of r-proteins (rpS13, rpS14, rpS5) was reduced close to background levels, though co-precipitation was still detectable (Fig. 4, compare 35S/32S signals in lanes 15, 19 and 23 with signals in lanes 16, 20 and 24, quantification of the signals (see Materials and Methods) indicated a reduction of purification efficiency by a factor of 10). As stated above, *E. coli* homologues of rpS13 and rpS14 belong to the central domain assembly tree which is implicated in folding of the SSU platform. RpS5 is located adjacent to rpS14 in the head - platform cleft and, S7, the *E. coli* homologue of rpS5, initiates the establishment of the SSU head domain assembly tree [37] (See also Fig. 1). To further characterize the impact of specific r-protein assembly events on the association of Noc4p with early pre-ribosomes we performed a semi-quantitative comparative proteome analysis of ribosome biogenesis factors co-purifying with TAP-tagged Noc4p from extracts of wildtype cells and cells depleted of either rpS5, rpS13, or rpS22 (Fig. 5). Co-purification of SSU processome components other than Nop14p/Noc5p was clearly reduced in cells depleted of rpS5 or rpS13 (see Fig. 5A, Fig. 5B and Fig. 5D). This suggested that association of Noc4p and its interaction partner Nop14p/Noc5p [24], [25] with SSU processome complexes lacking rpS5 or rpS13 was destabilized. In addition, these analyses confirmed the results of the (pre-)rRNA precipitation experiments (Fig. 4) that Noc4p-TAP continued to be stably incorporated in SSU processomes formed in the absence of rpS22 (see Fig. 5C and heatmap representation in Fig. 5D).

### Noc4p is required for efficient assembly of the 18S rRNA 3′ domain

**Figure 6:**
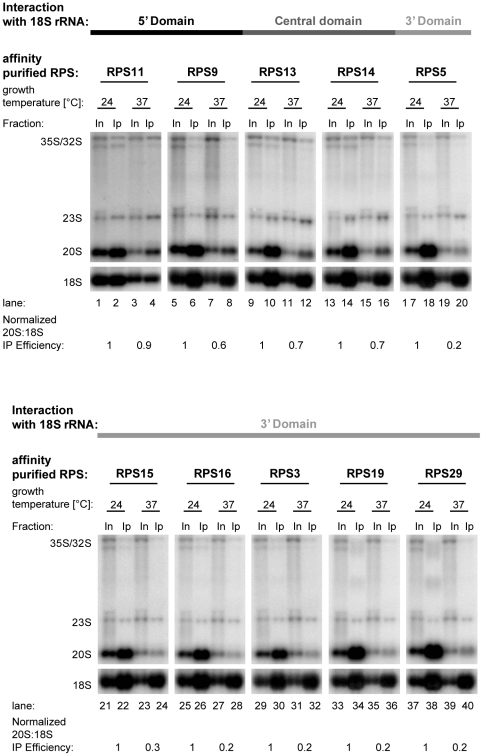
Analysis of (pre-) rRNAs co-purifying with Flag tagged r-proteins of the SSU in the yeast *noc4–8* mutant strain. The temperature sensitive *noc4–8* yeast mutant strain (TY40) was transformed with vectors supporting the constitutive expression of Flag tagged SSU r-proteins (see Fig. S4). Overnight cultures of transformants were grown for one generation time in full medium at 24°C to an OD of 0.4 and then cultivated for three hours in full medium at either permissive (24°C) or restrictive (37°C) temperature. The respective Flag-tagged r-protein was affinity purified from cellular extracts using anti-Flag M2 beads and co-purifying (pre-) rRNA species were analysed by Northern blotting using oligo 1819, which hybridizes in ribosomal precursor rRNAs between 18S and 5.8S rRNA sequences and detects 35S, 32S, 23S, and 20S pre-rRNAs (see Fig. S1). Oligo 205, which hybridizes within the 18S region, was used to detect 18S rRNA. Equal signal intensities of input (In) and affinity purified (IP) fractions correspond to 3% co-purification of the respective rRNA. The numbers in the lower panels indicate the efficiencies of 20S pre-rRNA purification divided by the efficiencies of 18S rRNA purification to normalize for possible over-all variations in the individual immuno-purification experiments. However, we note that the changes in 18S rRNA co-purification efficiencies between experiments performed with one transformant grown in permissive versus non-permissive conditions were in general below twenty percent. The numbers shown are the average of the results of two to four independent experiments and the values obtained with cells grown in permissive conditions were set to one.

The previous observations indicated that rpS5, rpS14, and rpS13 driven assembly and folding events in the SSU platform and head domain have an impact on the association of Noc4p with early pre-ribosomes. Interestingly, inactivation of Noc4p was shown to lead to an rRNA processing and transport phenotype closely resembling the one observed after shut down of rpS5 expression (compare Figs. 2 and 4, lane 23 with Fig. 6, lane 3, 7, 11, 15 and 19, see also references [24], [9]).

We investigated next, whether Noc4p might be involved in assembly of rpS5 or other SSU r-proteins. A temperature sensitive mutant of *NOC4*, *noc4–8* [24], was transformed with a collection of vectors supporting constitutive expression of Flag tagged r-proteins binding in all three major secondary structure domains of the 18S rRNA, respectively [10]. The constructs used complement the essential functions of the corresponding r-protein genes (data not shown). The strains were cultivated either at the permissive (24°C) or restrictive temperature (37°C). An anti-Flag immunoprecipitation was performed and (pre-) rRNA species co-purifying with the selected tagged r-proteins were analysed by Northern blotting. It was shown before that almost all r-proteins of the SSU show robust interactions both with mature small ribosomal subunits and with pre-ribosomes containing 20S pre-rRNA, the direct precursor of the mature 18S rRNA [10]. In contrast, their association with pre-ribosomal particles containing less matured rRNA species, like 23S or 35S pre-rRNAs, appeared to be less stable, indicating a gradual or stepwise tightening of r-protein interactions with pre-ribosomes during the course of *in vivo* maturation of the SSU [10]. Therefore, the efficiency of 20S pre-rRNA co-purification with the different r-proteins in the *noc4–8* mutant strain grown at permissive or restrictive conditions was taken as a measure for the successful establishment of a robust assembly state of the respective rpS in nascent ribosomes. The efficiency of individual immunoprecipitation reactions was internally controlled through the analysis of the precipitation efficiency of mature ribosomal subunits (containing the 18S rRNA) in which the Flag-tagged rpS variants were incorporated before shifting the cultures to restrictive conditions. Thus, the amount of precipitated 20S pre-rRNA could be normalised to the amount of precipitated mature 18S rRNA in individual immunoprecipitation experiments. As seen in Figure 6 (compare lanes 1, 5, 9, 13, 17, 21, 25, 29, 33 and 37 with lanes 3, 7, 11, 15, 19, 23, 27, 31, 35, and 39), inactivation of *noc4–8* resulted in a decreased level of 20S pre-rRNA, with the amount of early rRNA precursors accumulating in relation to 20S pre-rRNA. Inactivation of *noc4–8* led only to minor changes in the 20S pre-rRNA co-purification efficiency with Flag-tagged fusion r-proteins of the 18S rRNA 5′ (rpS9 and rpS11) and central domain (rpS13 and rpS14) (see Fig. 6, compare 18S rRNA and 20S pre-rRNA purification efficiency at permissive (24°C) and restrictive (37°C) conditions for tagged rpS9, rpS11, rpS13 and rpS14, quantification of the signals (see Materials and Methods) indicated 1.1 to 1.6 change in relative co-purification efficiency of 20S pre-rRNA versus 18S rRNA in permissive versus non-permissive condition,). In contrast, the co-purification efficiency of 20S pre-rRNA by Flag fusion r-proteins of the 3′ domain (rpS3, rpS5, rpS15, rpS16, rpS19, and rpS20) was significantly reduced at the restrictive temperature (see Fig. 6, compare 18S rRNA and 20S pre-rRNA purification efficiency at permissive (24°C) and restrictive (37°C) conditions for tagged rpS3, rpS5, rpS15 rpS16, rpS19 and rpS29, quantification of the signals indicated a 3.5 to 5.3 change in relative co-purification of 20S pre-rRNA versus 18S rRNA in permissive versus non-permissive conditions). No assembly defect of r-proteins was detectable by this approach in a wildtype strain cultivated at 37°C (data not shown).

In summary, these data indicated that Noc4p, whose stable association with early pre-ribosomes was suggested by the results of the previous experiments to be dependent on specific assembly events of both the SSU central (platform) and 3′ (head) domains, is itself required for efficient assembly of the SSU head domain.

## Discussion

The experiments presented indicate that members of the SSU processome sub-modules UTP-A and UTP-B continue to associate with early pre-ribosomes in strains disrupted in all tested r-proteins. Significantly, lack of assembly of r-proteins whose prokaryotic homologous proteins act according to *in vitro* reconstitution experiments as primary rRNA binders in five of six prokaryotic SSU assembly trees, did not detectably reduce the association of these SSU processome components with early pre-ribosomes. Accordingly, robust incorporation of the SSU processome sub-module UTP-A into pre-ribosomes does neither depend on the presence of other tested SSU processome components [26], [27] nor on the presence of any of the tested r-proteins (see Fig. 2). Altogether, this suggests that the UTP-A complex functions as transient primary binder in the hierarchy of eukaryotic SSU assembly.

Previous work indicated that the UTP-A sub-module in turn acts upstream of other SSU processome components including Noc4p [26]. The data presented here indicate that rpS5 and other r-proteins of the head domain are still able to interact *in vivo* to a certain extent with pre-ribosomes after inactivation of Noc4p. Nevertheless, establishment of more robust interactions of these r-proteins with rRNA required the presence of functional Noc4p. These observations reinforce the previous assumptions [38] that the combined action of SSU processome components plays a crucial role in facilitating such specific assembly events, as the conversion of initial, weak r-protein - pre-18S rRNA interactions into a stable complex.

Several hypotheses can be taken into consideration on how SSU processome components might drive specific assembly events.

Establishment of robust interactions of most SSU r-proteins with 18S rRNA precursors correlates in normal conditions with SSU processome dependent cleavage events in the 5′-ETS and the ITS-1 regions leading to 20S pre-rRNA ([10]; see also Figure 6, compare Flag-rpS co-purification efficiencies of 20S pre-RNA and 18S rRNA with the ones of 23S and 35S pre-rRNAs at permissive conditions). Hence, most SSU r-proteins show stabilized association with 20S pre-rRNA containing pre-ribosomes. SSU processome dependent pre-rRNA cleavage events leading to 20S pre-rRNA, in particular cleavage at site A_2_, were recently suggested to induce a conformational switch in pre-ribosomes [39] which might be a prerequisite for distinct r-protein assembly events. Nevertheless, the cleavages leading to 20S pre-rRNA seem not to be sufficient to drive progression of r-protein assembly since tightening of r-protein - pre-rRNA interactions is clearly affected on the level of the residual amounts of 20S pre-rRNA which is still produced in the absence of rpS5 expression [10], [40] or after inactivation of Noc4p (Fig. 6).

In contrast to r-proteins, SSU processome components interact strongly with largely un-processed nucleolar pre-rRNPs and weaker with more matured precursor particles (see for example Figs. 2 and 4, compare co-purification efficiencies of 20S pre-RNA with co-purification efficiencies of 23S and 35S pre-rRNAs). Early co-transcriptional and stable binding of SSU processome components could thereby inhibit *in vivo* formation of inter- or intramolecular contacts of rRNA precursors which interfere with the establishment of r-protein - pre-rRNA interactions. In agreement with this, the suggested SSU rRNA binding sites of the U3 snoRNA and snR30, another snoRNA essential for early pre-rRNA processing, are incompatible with the two major intramolecular rRNA contacts between the central and 5′ secondary structure domains observed in mature SSUs [31], [32], [41], [42]. Enzymatic activities, as for example RNA helicase activities, predicted for a few of the SSU processome components [43], [44], or potential direct contacts between SSU processome sub-modules and r-proteins might also contribute to stabilise transient r-protein-rRNA interactions [45]. Future *in vitro* studies on the impact of Noc4p and other SSU processome components on pre-rRNA folding and on the assembly of r-proteins should help to understand in more detail the mode of their action in early steps of eukaryotic SSU maturation.

A subset of SSU processome components (Rrp7p/Utp22p group in Fig. 3B), including the RNA helicase Rok1p and the UTP-C sub-module members Rrp7p and Utp22p were identified here to be specifically affected in their association with early SSU precursors after *in vivo* depletion of rpS13 and rpS14. The *E. coli* homologues of rpS13 and rpS14, S15 and S11, are primary and tertiary binder of one of the central domain assembly trees important for folding of the SSU platform. Inactivation or *in vivo* depletion of Rok1p, Rrp7p, Utp22p, rpS13, or rpS14 (and other central domain binders as rpS1 and rpS27) leads to similar early 18S pre-rRNA processing phenotypes [9], [46]–[48]. Interestingly, overexpression of rpS27, which binds in the SSU rRNA central domain adjacent to rpS13 [31], [32], rescues the lethal phenotype of yeast *rrp7* deletion mutants [48]. In addition, *in vivo* depletion of the helicase Rok1p was shown to affect specifically the pre-rRNP association of snR30 [49]. SnR30 is one of the three small nucleolar RNAs essential for early steps of rRNA maturation [50] which was recently shown to bind *in vivo* to sequences of the eukaryote specific expansion segment 6 in the rRNA central domain [42]. These data further indicate a specific functional link between the SSU central domain assembly state and early SSU precursor interactions of factors as Rok1p and UTP-C sub-module members.

Other SSU processome components (Noc4p/Nop14p group in Fig. 3B) were affected in their association with early SSU precursors not only by *in vivo* depletion of rpS13 and of rpS14, but also after shut down of RPS5 expression. RpS5 binds in the SSU head domain adjacent to the platform constituent rpS14. Its *E. coli* homologue S7 is the primary binder of the *in vitro* assembly tree of SSU head domain r-proteins. Consistent with this, yeast rpS5 is required for efficient *in vivo* assembly of the eukaryotic SSU head constituents rpS3, rpS10, rpS15, rpS16, rpS19, rpS20, rpS28 and rpS29 [10]. Several SSU processome components whose association with early SSU precursors were affected by rpS5 depletion were shown previously to interact with each other or with constituents of the SSU head domain. Interactions between Bms1p and Rcl1p were observed *in vitro* [51], [52] and in two hybrid assays [20] and were furthermore indicated by *ex-vivo* co-purification experiments [17], [27]. Large scale analyses revealed genetic interactions between Noc4p and Utp30p [53] and between Utp30p and Rrp7p [54]. Moreover, Noc4p forms a salt resistant protein complex with Nop14p [25], [55]. Nop14p interacts in two hybrid assays with Emg1p/Nep1p [56], a pseudouridine N1-methyltransferase required for methylation of pseudouridine 1191 in the yeast SSU head domain [57]. The lethal phenotype of an *emg1* deletion mutant strain was shown to be rescued by overexpression of RPS19B [45], whose gene product rpS19 is stably incorporated into the SSU head domain in a Noc4p (see above) and rpS5 dependent way [10]. Finally, pre-rRNA interaction sites and localization of Enp1p were recently mapped in the SSU rRNA 3′ domain [58], [59] and Enp1-TAP fusion proteins showed reduced efficiency in co-purification of early pre-ribosomal particles after depletion of Noc4p (see Fig. S5, note that Noc4p depletion did not significantly affect the association of Utp4p, Pwp2p, Utp22p or Imp3p with early pre-ribosomes). In conclusion, these data reinforce the existence of a functional interaction network among members of the Noc4p/Nop14p group (Fig. 3B) and SSU head domain constituents.

Interestingly, Noc4p was affected in its association with early pre-ribosome by *in vivo* depletion of rpS5 and the central domain binders rpS13 and rpS14, being itself required for r-protein assembly events in the SSU head domain. One straight forward interpretation of these observations is that a distinct central domain assembly state has to be established to allow efficient recruitment of Noc4p to pre-ribosomes. Noc4p, potentially together with other factors as Nop14p, Emg1p and Enp1p, could then facilitate in a cooperative way downstream r-protein assembly events in the SSU head domain. In such a scenario, the SSU processome component Noc4p coordinates early steps of *in vivo* folding and assembly of the central and the 3′ major 18S rRNA secondary structure domains thereby providing a quality control checkpoint in the process of eukaryotic SSU assembly.

## Materials and Methods

### Yeast strains and microbiological procedures

Yeast strains used in this study are listed in Figure S2. To construct strains expressing endogenously TAP-tagged SSU processome factors (Utp4p, Pwp2p, Noc4p, Enp1p, Utp22p, Imp3p) the TAP-URA3-cassette on plasmid pBS1539 was PCR-amplified using the respective primers given in Figure S3 [61]. The purified PCR product was transformed into competent yeast cells [62] and the correct genomic integration of the TAP-URA3 cassette was verified by selection for uracil prototrophy on appropriate minimal medium (SCG-URA) and Western blot analysis. Description of yeast strains, oligos and plasmids used in this study are indicated in Figures S2, S3, S4.

The strains conditionally expressing certain SSU r-protein genes were cultivated at 30°C in YPG (1% yeast extract, 2% bacto peptone, 2% galactose); expression of the respective genes was shut down by shift to YPD (1% yeast extract, 2% bacto peptone, 2% glucose) for 4 hours at 30°C.

The temperature sensitive *noc4–8* strain was transformed with plasmids coding for the respective SSU proteins fused to the FLAG tag (see Fig. S4) and cultivated overnight at 24°C in appropriate minimal medium (SCD-Ura). After overnight cultivation the culture was diluted in YPD and grown for 3 h at 24°C. The culture was then split and one part was incubated for 3 h at 24°C whereas the other part was incubated for 3 h at 37°C.

### Northern Blotting analyses

RNA was extracted by hot phenol-chloroform treatment [24] and resolved on denaturating agarose gels (1.3% agarose (Invitrogen), 2% formaldehyde; 0.1 mg/ml ethidium bromide; 1× MOPS buffer (20 mM MOPS, 2 mM NaOAc, 1 mM EDTA, pH7)) as described in [63]. Gels were run for 14–16 h at 40 V in electrophoresis buffer (1× MOPS buffer, 2% formaldehyde). The transfer from the gel onto the positively charged membrane (Positive TM, MP-Biomedicals) was performed in 10× SSC buffer by applying a vacuum of 5 bar for 90 min using a vacuum blotter (Biorad). Hybridization was performed in 50% formamide; 5× SSC; 0,5% SDS; 5× Denhards solution at 30°C. The sequence identity of oligos used for detection of different (pre-)rRNAs is indicated in Figure S3. The blots were washed twice for 15 min with 2× SSC at 30°C. Labelled rRNA signals were detected by exposing the membrane to a Phosphoimager screen and using a Phosphor Imager FLA3000 (Fujifilm). Data were quantified using MultiGauge V3.0 (Fujifilm).

### Western Blotting analyses

Expression and precipitation levels of TAP-tagged biogenesis factors in the conditional rpS strains were determined by Western blot analysis. Same amounts of whole cell extracts, were analyzed using PAP visualisation reagent (DakoCytomation, Z 0113) in a dilution of 1∶3000 for detection of the TAP-tag. Noc4p was detected by a rat monoclonal anti-Noc4p antibody. Protein signals were visualised by chemiluminescence using a Fluorescence Image Reader LAS3000 (Fujifilm). Data were quantified using MultiGauge V3.0 (Fujifilm).

### Co-immunoprecipitation of (pre-) rRNPs using IgG or anti-FLAG antibody coupled sepharose beads

Affinity purification of tagged proteins on respective IgG or anti FLAG antibody coupled sepharose beads was performed as described in [40] with the following modifications. The cell pellet corresponding to 100 ml yeast culture with OD_600_ = 0.8–1.0 was resuspended in 500 µl cold A200 buffer (20 mM Tris–HCl pH 8, 200 mM KCl, 5 mM MgOAc, 0.2% Triton X-100, 1 mM DTT, 2 mM Benzamidine, 1 mM PMSF) containing 0.04 U/µl RNasin. A cell lysate was prepared by vigorous shaking of the cell suspension with 1.4 ml glass beads (Ø 0.75–1 mm) in a IKA-Vibrax VXR shaker for 20 min, followed by 2 min on ice and another 20 min shaking in the Vibrax. The cell lysate was cleared from cell debris by two centrifugation steps, 1×5 min at 14000 rpm and 1×10 min at 14000 rpm. The protein concentration of the cleared lysate was determined using the Bradford assay. 6 mg of whole protein extract was incubated with 120 µl of equilibrated (3× washing with A200 buffer) IgG coupled sepharose beads slurry (Amersham) and rotated for 1.5 h at 4°C. The beads were washed 7 times (1×1 ml, 5×2 ml and 1×10 ml) with cold A200 buffer in a 10 ml column. For the precipitation of TAP tagged biogenesis factors the washed beads were split and 1/6 was used for protein analysis by Western blotting, whereas 5/6 was used for RNA analysis by Northern blotting.

Co-immunoprecipitation of (pre-)rRNA using 90 ul of anti-Flag M2 beads slurry (Sigma) was performed essentially the same as with IgG coupled sepharose beads. All washed beads were used for RNA analysis by Northern blotting.

### Affinity purification using IgG coupled magnetic beads

Affinity purification of pre-ribosomal particles was performed essentially as described in [64] with the following modifications. The cell pellet corresponding to 2.5 l yeast culture with OD_600_ = 0.8–1.0 was resuspended in 1.5 ml of cold MB buffer (20 mM Tris–HCl pH 8, 200 mM KCl, 5 mM MgOAc, 2 mM Benzamidine, 1 mM PMSF,1 mM DTT and 0.04 U/µl RNasin) per gramm of cell pellet. 800 µl of this cell suspension was added to 1.4 ml glass beads (Ø 0.75–1 mm) and divided into 2 ml reaction tubes. A cell lysate was prepared by vigorous shaking of the cell suspension in a IKA-Vibrax VXR shaker at 4°C for 20 min, followed by 2 min on ice. This procedure was repeated twice. The cell lysate was cleared from cell debris by two centrifugation steps, 1×5 min at 14000 rpm and 1×10 min at 14000 rpm. The protein concentration of the cleared lysate was determined using the Bradford assay. Triton X-100 (0.5%) and Tween 20 (0.1%) was added to the cell lysate. The whole amount of cell lysate (typically 2.0–2.4 ml with 120–180 mg of total protein) was incubated for 1 hour at 4°C with 250 µl of IgG (rabbit serum, I5006-100MG, Sigma) coupled magnetic beads slurry (1 µm BcMag, FC-102, Bioclone) equilibrated in MB buffer containing 0.5% Triton X-100 and 0.1% Tween. The beads were washed four times with 700 µl cold MB buffer with 0.5% Triton X-100 and 0.1% Tween 20 and were then washed two times with AC buffer (100 mM NH_4_OAc pH 7.4, 0.1 mM MgCl_2_) to remove remaining salt from the sample. Bound proteins were eluted two times with 500 µl of freshly prepared 500 mM NH_4_OH solution for 20 min at RT. Both eluate fractions were pooled and lyophilised over night.

### Comparative MALDI TOF/TOF analyses

The lyophilised protein samples were resuspended in 20 µl dissolution buffer (iTRAQ™ labelling kit, Invitrogen) and reduced with 5 mM Tris-(2-carboxyethyl)phosphine at 60°C for 1 h. Cysteins were blocked with 10 mM methyl-methanethiosulfonate (MMTS) at room temperature for 10 min. After trypsin digestion for 20 h at 37°C, tryptic peptides of the purifications of interest were labelled with different combinations of the four iTRAQ™ reagents according to the manufacturer (Invitrogen). The differentially labelled peptides were combined and lyophilised [35], [36].

The combined differently labelled peptides were dissolved for 2 h in 0.1%TFA and loaded on a nano-flow HPLC-system (Dionex) harbouring a C18-Pep-Mep column (LC-Packings). The peptides were separated by a gradient of 5% to 95% of buffer B (80% acetonitrile/0.05% TFA) and fractions were mixed with 5 volumes of CHCA (alpha-cyano-4-hydroxy cinnamic acid; Sigma) matrix (2 mg/ml in 70% acetonitrile/0.1%TFA) and spotted online via the Probot system (Dionex) on a MALDI-target.

MS/MS analyses were performed on an Applied Biosystems 4700 or 4800 Proteomics Analyzer MALDI-TOF/TOF mass spectrometer operated in positive ion reflector mode and evaluated by searching the NCBInr protein sequence database with the Mascot search engine (Matrix Science) implemented in the GPS Explorer software (Applied Biosystems). Laser intensity was adjusted due to laser condition and sample concentration. The eight most intense peptide peaks per spot detected in the MS mode were further fragmented yielding the respective MS/MS spectra.

The peak area for iTRAQ™ reporter ions were interpreted and corrected by the GPS-Explorer software (Applied Biosystems) and Excel (Microsoft). An iTRAQ ratio average of all peptides of a given protein was calculated. Hierarchical clustering analysis of datasets derived from several experiments was done with cluster 3.0 software [65] using the “log2 transform data” and the “median center arrays” settings for data adjustment and the euclidean distance and centroid linkage settings for gene and array clustering. Data were normalized before cluster analyses by setting the respective Utp4p-TAP iTRAQ ratios to one. Java Treeview was used for cluster visualization (see http://www.eisenlab.org/eisen/?page_id=42).

## Supporting Information

Figure S1 **Schematic view of the processing of SSU rRNA precursors in** ***S. cerevisiae*** **.** The upper panel shows a schematic drawing of the primary transcript including the 18S, 5.8S, and 25S rRNA genes, the external transcribed spacers (5′ ETS and 3′ ETS), and the internal transcribed sequences (ITS-1 and ITS-2). In addition, the known processing sites are depicted. Processing starts at site B_0_ yielding the first detectable rRNA transcript, the 35S pre-rRNA. The processing steps marked by big arrows indicate the major processing pathway of the SSU. Cleavage at sites A_0_ and A_1_ generates the 33S and 32S rRNA, respectively (not shown) and cleavage at site A_2_ separates the precursor of the SSU (20S pre-rRNA) from the precursor of the LSU (27SA_2_ pre-rRNA, not shown). In a minor processing pathway, cleavage is initiated in the ITS-1, yielding the 23S and 27SA_3_ (not shown) pre-rRNAs. Further processing at sites A_0_, A_1_, and A_2_ results in the 22S, 21S, and 20S pre-rRNAs, respectively. 23S, 22S, and 21S pre-rRNAs also accumulate in mutants in which processing at sites A_0_, A_1_, and A_2_ is fully or partly inhibited. The hybridisation sites of probes 205 (18S) and 1819 (ITS-1) are depicted.
(TIF)

Figure S2 **Yeast strains used in this study.**
(PDF)

Figure S3 **Oligos used in this study.**
(PDF)

Figure S4 **Plasmids used in this study.**
(PDF)

Figure S5 **Analysis of (pre-) rRNAs co-purifying with UTP-A, UTP-B, or UTP-C SSU processome components and with Enp1p after** ***in vivo*** **depletion of Noc4p.** The yeast strains TY1903, TY1904, TY1905, TY1906, and TY2112 expressing chromosome encoded TAP tagged Utp4p, Pwp2p, Utp22p, Imp3p, and Enp1p, respectively, and carrying in addition a galactose inducible conditional allele of NOC4 were either cultivated in medium containing galactose as carbon source (on) or were transferred to glucose containing medium (off) and cultivated for additional 16 hours. TAP fusion proteins were affinity purified from corresponding cellular extracts using IgG coupled Sepharose beads. *In vivo* depletion of Noc4p and the amount of the purified bait proteins were monitored by Western blotting (middle and lower panels) and co-purified pre-rRNA species were analysed by Northern blotting (upper panel) using oligo 1819, which hybridizes in ribosomal precursor rRNAs between 18S and 5.8S rRNA sequences and detects 35S, 32S, 23S, and 20S pre-rRNAs (see Fig. S1). Equal signal intensities of input (In) and beads (IP) fractions in Northern blots correspond to 1% co-precipitation of the respective rRNA. Efficiencies of 35S pre-rRNA purification normalized to the values obtained for cells grown in permissive conditions are indicated in the lower panel. For the Western blot analyses equal signal intensities of input (In) and beads (IP) correspond to 20% precipitation of the TAP-tagged bait protein.
(TIF)

## References

[1] Held WA, Ballou B, Mizushima S, Nomura M (1974). Assembly mapping of 30 S ribosomal proteins from Escherichia coli. Further studies.. J Biol Chem.

[2] Herold M, Nierhaus KH (1987). Incorporation of six additional proteins to complete the assembly map of the 50 S subunit from Escherichia coli ribosomes.. J Biol Chem.

[3] Nierhaus KH (1991). The assembly of prokaryotic ribosomes.. Biochimie.

[4] Sykes MT, Williamson JR (2009). A complex assembly landscape for the 30S ribosomal subunit.. Annu Rev Biophys.

[5] Weitzmann CJ, Cunningham PR, Nurse K, Ofengand J (1993). Chemical evidence for domain assembly of the Escherichia coli 30S ribosome.. FASEB J.

[6] Samaha RR, O'Brien B, O'Brien TW, Noller HF (1994). Independent in vitro assembly of a ribonucleoprotein particle containing the 3′ domain of 16S rRNA.. Proc Natl Acad Sci USA.

[7] Agalarov SC, Zheleznyakova EN, Selivanova OM, Zheleznaya LA, Matvienko NI (1998). In vitro assembly of a ribonucleoprotein particle corresponding to the platform domain of the 30S ribosomal subunit.. Proc Natl Acad Sci USA.

[8] Adilakshmi T, Bellur DL, Woodson SA (2008). Concurrent nucleation of 16S folding and induced fit in 30S ribosome assembly.. Nature.

[9] Ferreira-Cerca S, Pöll G, Gleizes P-E, Tschochner H, Milkereit P (2005). Roles of eukaryotic ribosomal proteins in maturation and transport of pre-18S rRNA and ribosome function.. Mol Cell.

[10] Ferreira-Cerca S, Pöll G, Kühn H, Neueder A, Jakob S (2007). Analysis of the in vivo assembly pathway of eukaryotic 40S ribosomal proteins.. Mol Cell.

[11] Karbstein K (2011). Inside the 40S ribosome assembly machinery.. Curr Opin Chem Biol.

[12] Henras AK, Soudet J, Gérus M, Lebaron S, Caizergues-Ferrer M (2008). The post-transcriptional steps of eukaryotic ribosome biogenesis.. Cell Mol Life Sci.

[13] Udem SA, Warner JR (1972). Ribosomal RNA synthesis in Saccharomyces cerevisiae.. J Mol Biol.

[14] Trapman J, Retel J, Planta RJ (1975). Ribosomal precursor particles from yeast.. Exp Cell Res.

[15] Dragon F, Gallagher JE, Compagnone-Post PA, Mitchell BM, Porwancher KA (2002). A large nucleolar U3 ribonucleoprotein required for 18S ribosomal RNA biogenesis.. Nature.

[16] Grandi P, Rybin V, Bassler J, Petfalski E, Strauss D (2002). 90S pre-ribosomes include the 35S pre-rRNA, the U3 snoRNP, and 40S subunit processing factors but predominantly lack 60S synthesis factors.. Mol Cell.

[17] Krogan NJ, Peng W-T, Cagney G, Robinson MD, Haw R (2004). High-definition macromolecular composition of yeast RNA-processing complexes.. Mol Cell.

[18] Gallagher JEG, Dunbar DA, Granneman S, Mitchell BM, Osheim Y (2004). RNA polymerase I transcription and pre-rRNA processing are linked by specific SSU processome components.. Genes Dev.

[19] Dosil M, Bustelo XR (2004). Functional characterization of Pwp2, a WD family protein essential for the assembly of the 90 S pre-ribosomal particle.. J Biol Chem.

[20] Wegierski T, Billy E, Nasr F, Filipowicz W (2001). Bms1p, a G-domain-containing protein, associates with Rcl1p and is required for 18S rRNA biogenesis in yeast.. RNA.

[21] Granneman S, Kudla G, Petfalski E, Tollervey D (2009). Identification of protein binding sites on U3 snoRNA and pre-rRNA by UV cross-linking and high-throughput analysis of cDNAs.. Proc Natl Acad Sci USA.

[22] Venema J, Vos HR, Faber AW, van Venrooij WJ, Raué HA (2000). Yeast Rrp9p is an evolutionarily conserved U3 snoRNP protein essential for early pre-rRNA processing cleavages and requires box C for its association.. RNA.

[23] Granneman S, Gallagher JE, Vogelzangs J, Horstman W, van Venrooij WJ (2003). The human Imp3 and Imp4 proteins form a ternary complex with hMpp10, which only interacts with the U3 snoRNA in 60–80S ribonucleoprotein complexes.. Nucleic Acids Res.

[24] Milkereit P, Strauss D, Bassler J, Gadal O, Kühn H (2003). A Noc complex specifically involved in the formation and nuclear export of ribosomal 40 S subunits.. J Biol Chem.

[25] Kühn H, Hierlmeier T, Merl J, Jakob S, Aguissa-Touré A-H (2009). The Noc-domain containing C-terminus of Noc4p mediates both formation of the Noc4p-Nop14p submodule and its incorporation into the SSU processome.. PLoS ONE.

[26] Pérez-Fernández J, Román A, De Las Rivas J, Bustelo XR, Dosil M (2007). The 90S preribosome is a multimodular structure that is assembled through a hierarchical mechanism.. Mol Cell Biol.

[27] Pérez-Fernández J, Martín-Marcos P, Dosil M (2011). Elucidation of the assembly events required for the recruitment of Utp20, Imp4 and Bms1 onto nascent pre-ribosomes.. Nucleic Acids Res.

[28] Turner AJ, Knox AA, Prieto J-L, McStay B, Watkins NJ (2009). A novel small-subunit processome assembly intermediate that contains the U3 snoRNP, nucleolin, RRP5, and DBP4.. Mol Cell Biol.

[29] Chooi WY, Leiby KR (1981). An electron microscopic method for localization of ribosomal proteins during transcription of ribosomal DNA: a method for studying protein assembly.. Proc Natl Acad Sci USA.

[30] Wery M, Ruidant S, Schillewaert S, Leporé N, Lafontaine DLJ (2009). The nuclear poly(A) polymerase and Exosome cofactor Trf5 is recruited cotranscriptionally to nucleolar surveillance.. RNA.

[31] Ben-Shem A, Jenner L, Yusupova G, Yusupov M (2010). Crystal structure of the eukaryotic ribosome.. Science.

[32] Rabl J, Leibundgut M, Ataide SF, Haag A, Ban N (2011). Crystal Structure of the Eukaryotic 40S Ribosomal Subunit in Complex with Initiation Factor 1.. Science.

[33] Rigaut G, Shevchenko A, Rutz B, Wilm M, Mann M (1999). A generic protein purification method for protein complex characterization and proteome exploration.. Nat Biotechnol.

[34] O'Donohue M-F, Choesmel V, Faubladier M, Fichant G, Gleizes P-E (2010). Functional dichotomy of ribosomal proteins during the synthesis of mammalian 40S ribosomal subunits.. J Cell Biol.

[35] Ross PL, Huang YN, Marchese JN, Williamson B, Parker K (2004). Multiplexed protein quantitation in Saccharomyces cerevisiae using amine-reactive isobaric tagging reagents.. Mol Cell Proteomics.

[36] Merl J, Jakob S, Ridinger K, Hierlmeier T, Deutzmann R (2010). Analysis of ribosome biogenesis factor-modules in yeast cells depleted from pre-ribosomes.. Nucleic Acids Res..

[37] Nowotny V, Nierhaus KH (1988). Assembly of the 30S subunit from Escherichia coli ribosomes occurs via two assembly domains which are initiated by S4 and S7.. Biochemistry.

[38] Bernstein KA, Gallagher JEG, Mitchell BM, Granneman S, Baserga SJ (2004). The small-subunit processome is a ribosome assembly intermediate.. Eukaryotic Cell.

[39] Lamanna AC, Karbstein K (2011). An RNA conformational switch regulates pre-18S rRNA cleavage.. J Mol Biol.

[40] Neueder A, Jakob S, Pöll G, Linnemann J, Deutzmann R (2010). A local role for the small ribosomal subunit primary binder rpS5 in final 18S rRNA processing in yeast.. PLoS ONE.

[41] Hughes JM (1996). Functional base-pairing interaction between highly conserved elements of U3 small nucleolar RNA and the small ribosomal subunit RNA.. J Mol Biol.

[42] Fayet-Lebaron E, Atzorn V, Henry Y, Kiss T (2009). 18S rRNA processing requires base pairings of snR30 H/ACA snoRNA to eukaryote-specific 18S sequences.. EMBO J.

[43] Strunk BS, Karbstein K (2009). http://www.ncbi.nlm.nih.gov/pubmed/19850913.

[44] Granneman S, Bernstein KA, Bleichert F, Baserga SJ (2006). Comprehensive mutational analysis of yeast DEXD/H box RNA helicases required for small ribosomal subunit synthesis.. Mol Cell Biol.

[45] Buchhaupt M, Meyer B, Kötter P, Entian K-D (2006). Genetic evidence for 18S rRNA binding and an Rps19p assembly function of yeast nucleolar protein Nep1p.. Mol Genet Genomics.

[46] Venema J, Bousquet-Antonelli C, Gelugne JP, Caizergues-Ferrer M, Tollervey D (1997). Rok1p is a putative RNA helicase required for rRNA processing.. Mol Cell Biol.

[47] Peng WT, Robinson MD, Mnaimneh S, Krogan NJ, Cagney G (2003). A panoramic view of yeast noncoding RNA processing.. Cell.

[48] Baudin-Baillieu A, Tollervey D, Cullin C, Lacroute F (1997). Functional analysis of Rrp7p, an essential yeast protein involved in pre-rRNA processing and ribosome assembly.. Mol Cell Biol.

[49] Bohnsack MT, Kos M, Tollervey D (2008). Quantitative analysis of snoRNA association with pre-ribosomes and release of snR30 by Rok1 helicase.. EMBO Rep.

[50] Morrissey JP, Tollervey D (1993). Yeast snR30 is a small nucleolar RNA required for 18S rRNA synthesis.. Mol Cell Biol.

[51] Karbstein K, Jonas S, Doudna JA (2005). An essential GTPase promotes assembly of preribosomal RNA processing complexes.. Mol Cell.

[52] Karbstein K, Doudna JA (2006). GTP-dependent formation of a ribonucleoprotein subcomplex required for ribosome biogenesis.. J Mol Biol.

[53] Costanzo M, Baryshnikova A, Bellay J, Kim Y, Spear ED (2010). The Genetic Landscape of a Cell.. Science.

[54] Wilmes GM, Bergkessel M, Bandyopadhyay S, Shales M, Braberg H (2008). A genetic interaction map of RNA-processing factors reveals links between Sem1/Dss1-containing complexes and mRNA export and splicing.. Mol Cell.

[55] Milkereit P, Strauss D, Bassler J, Gadal O, Kühn H (2003). A Noc complex specifically involved in the formation and nuclear export of ribosomal 40 S subunits.. J Biol Chem.

[56] Liu PC, Thiele DJ (2001). Novel stress-responsive genes EMG1 and NOP14 encode conserved, interacting proteins required for 40S ribosome biogenesis.. Mol Biol Cell.

[57] Meyer B, Wurm JP, Kötter P, Leisegang MS, Schilling V (2011). The Bowen-Conradi syndrome protein Nep1 (Emg1) has a dual role in eukaryotic ribosome biogenesis, as an essential assembly factor and in the methylation of Ψ1191 in yeast 18S rRNA.. Nucleic Acids Res.

[58] Strunk BS, Loucks CR, Su M, Vashisth H, Cheng S (2011). Ribosome assembly factors prevent premature translation initiation by 40S assembly intermediates.. Science.

[59] Granneman S, Petfalski E, Swiatkowska A, Tollervey D (2010). Cracking pre-40S ribosomal subunit structure by systematic analyses of RNA-protein cross-linking.. EMBO J.

[60] Recht MI, Williamson JR (2004). RNA tertiary structure and cooperative assembly of a large ribonucleoprotein complex.. J Mol Biol.

[61] Puig O, Caspary F, Rigaut G, Rutz B, Bouveret E (2001). The tandem affinity purification (TAP) method: a general procedure of protein complex purification.. Methods.

[62] Knop M, Siegers K, Pereira G, Zachariae W, Winsor B (1999). Epitope tagging of yeast genes using a PCR-based strategy: more tags and improved practical routines.. Yeast.

[63] Sambrook J, Fritsch EF, Maniatis T (1989). Molecular Cloning: A Laboratory Manual..

[64] Oeffinger M, Wei KE, Rogers R, DeGrasse JA, Chait BT (2007). Comprehensive analysis of diverse ribonucleoprotein complexes.. Nat Methods.

[65] Eisen MB, Spellman PT, Brown PO, Botstein D (1998). Cluster analysis and display of genome-wide expression patterns.. Proceedings of the National Academy of Sciences.

