# Supplementary figures and images for: Interrelationships between Yeast Ribosomal Protein Assembly Events and Transient Ribosome Biogenesis Factors Interactions in Early Pre-Ribosomes
Source: PLoS One. 2012 Mar 14;7(3):e32552. doi: 10.1371/journal.pone.0032552 (PMC3303783; doi:10.1371/journal.pone.0032552)

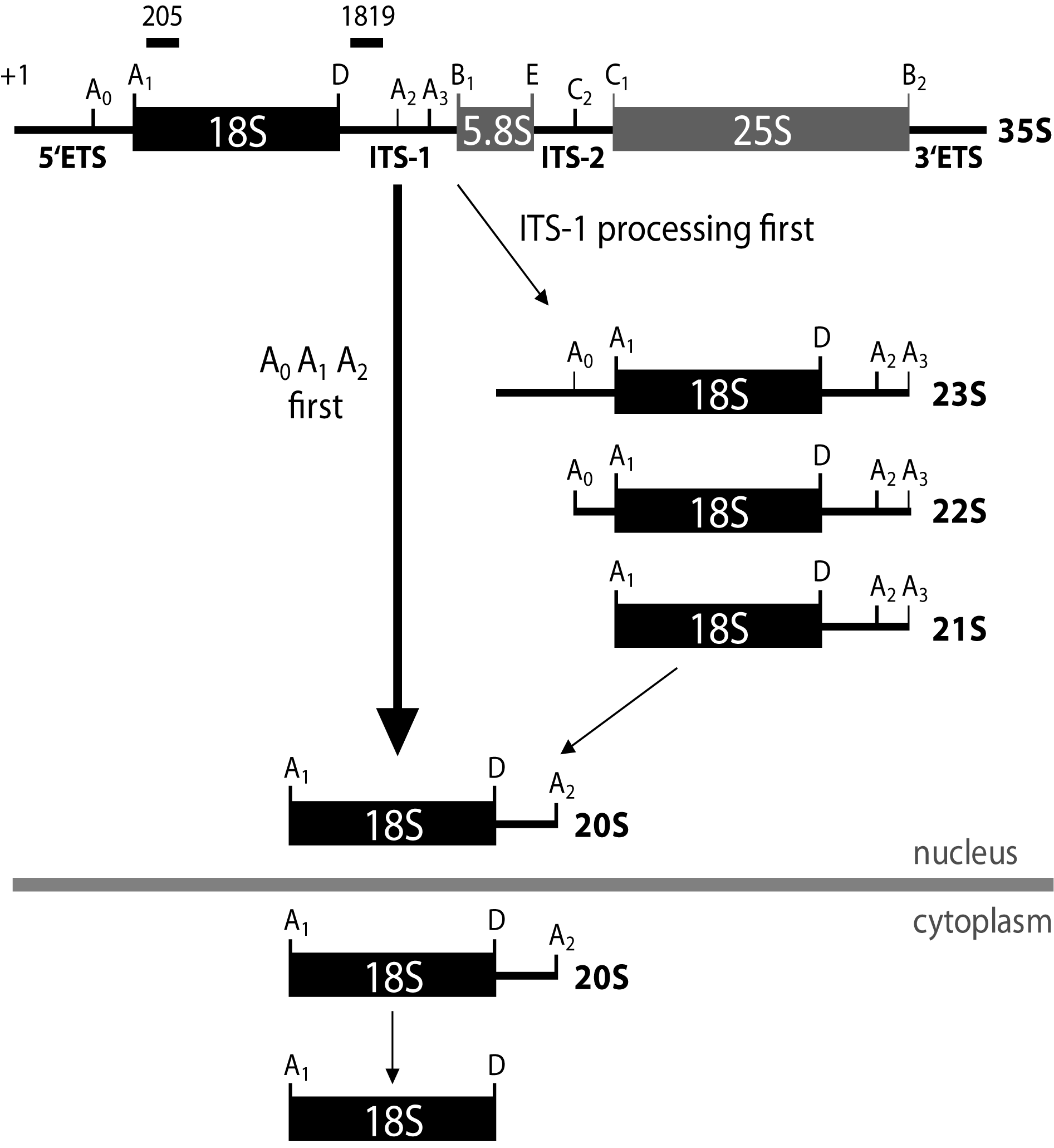

Supplement: Figure S1 — Schematic view of the processing of SSU rRNA precursors in S. cerevisiae . The upper panel shows a schematic drawing of the primary transcript including the 18S, 5.8S, and 25S rRNA genes, the external transcribed spacers (5′ ETS and 3′ ETS), and the internal transcribed sequences (ITS-1 and ITS-2). In addition, the known processing sites are depicted. Processing starts at site B0 yielding the first detectable rRNA transcript, the 35S pre-rRNA. The processing steps marked by big arrows indicate the major processing pathway of the SSU. Cleavage at sites A0 and A1 generates the 33S and 32S rRNA, respectively (not shown) and cleavage at site A2 separates the precursor of the SSU (20S pre-rRNA) from the precursor of the LSU (27SA2 pre-rRNA, not shown). In a minor processing pathway, cleavage is initiated in the ITS-1, yielding the 23S and 27SA3 (not shown) pre-rRNAs. Further processing at sites A0, A1, and A2 results in the 22S, 21S, and 20S pre-rRNAs, respectively. 23S, 22S, and 21S pre-rRNAs also accumulate in mutants in which processing at sites A0, A1, and A2 is fully or partly inhibited. The hybridisation sites of probes 205 (18S) and 1819 (ITS-1) are depicted. (TIF) [file pone.0032552.s001.tif]

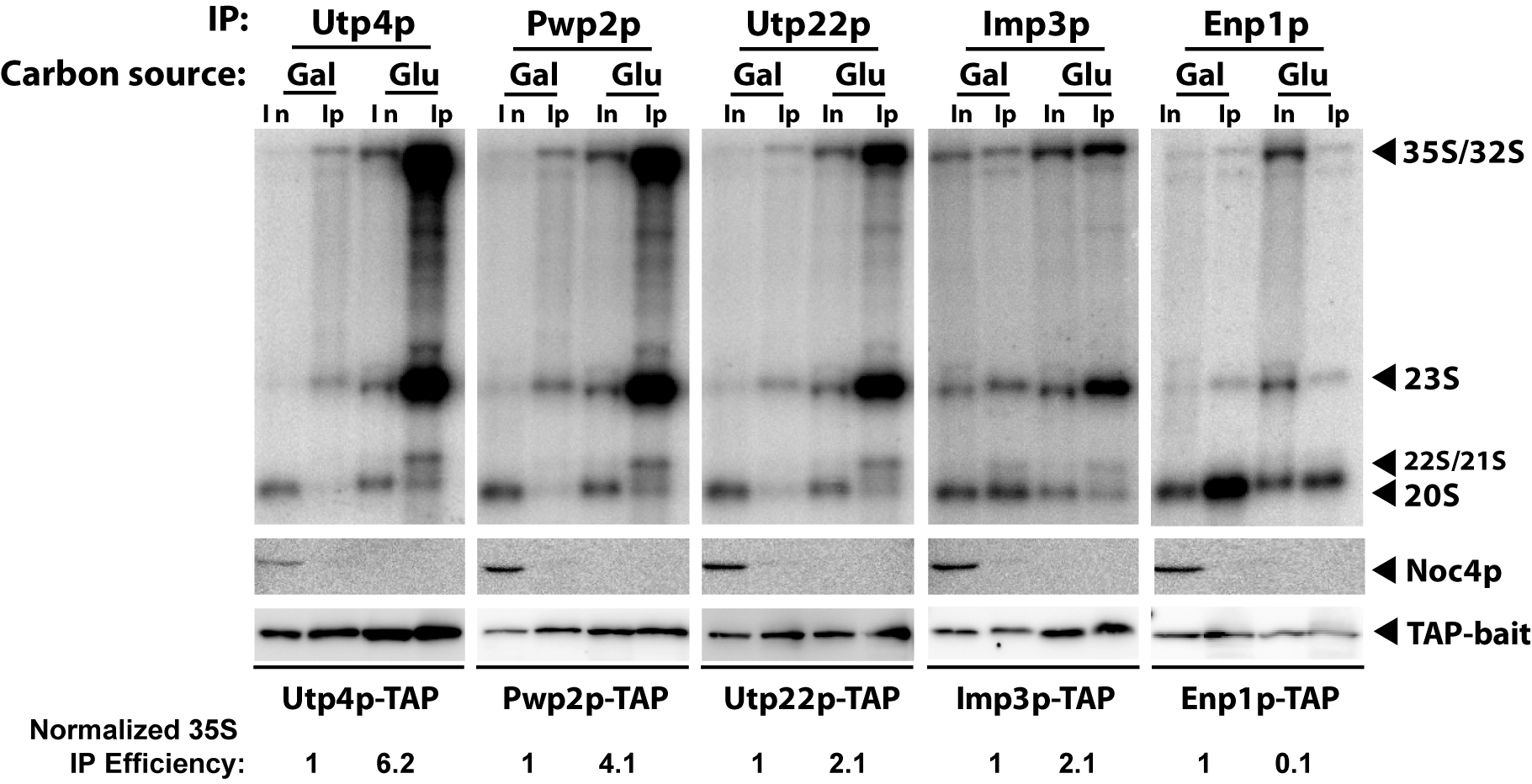

Supplement: Figure S5 — Analysis of (pre-) rRNAs co-purifying with UTP-A, UTP-B, or UTP-C SSU processome components and with Enp1p after in vivo depletion of Noc4p. The yeast strains TY1903, TY1904, TY1905, TY1906, and TY2112 expressing chromosome encoded TAP tagged Utp4p, Pwp2p, Utp22p, Imp3p, and Enp1p, respectively, and carrying in addition a galactose inducible conditional allele of NOC4 were either cultivated in medium containing galactose as carbon source (on) or were transferred to glucose containing medium (off) and cultivated for additional 16 hours. TAP fusion proteins were affinity purified from corresponding cellular extracts using IgG coupled Sepharose beads. In vivo depletion of Noc4p and the amount of the purified bait proteins were monitored by Western blotting (middle and lower panels) and co-purified pre-rRNA species were analysed by Northern blotting (upper panel) using oligo 1819, which hybridizes in ribosomal precursor rRNAs between 18S and 5.8S rRNA sequences and detects 35S, 32S, 23S, and 20S pre-rRNAs (see Fig. S1). Equal signal intensities of input (In) and beads (IP) fractions in Northern blots correspond to 1% co-precipitation of the respective rRNA. Efficiencies of 35S pre-rRNA purification normalized to the values obtained for cells grown in permissive conditions are indicated in the lower panel. For the Western blot analyses equal signal intensities of input (In) and beads (IP) correspond to 20% precipitation of the TAP-tagged bait protein. (TIF) [file pone.0032552.s005.tif]
