# Supplementary material for: Interrelationships between Yeast Ribosomal Protein Assembly Events and Transient Ribosome Biogenesis Factors Interactions in Early Pre-Ribosomes
Source: PLoS One. 2012 Mar 14;7(3):e32552. doi: 10.1371/journal.pone.0032552 (PMC3303783; doi:10.1371/journal.pone.0032552)
Supplement: Figure S2 — Yeast strains used in this study. (PDF) [file pone.0032552.s002.pdf]

Fig. S2 Yeast strains used in this study

Name	TY	Genotype	Origin
noc4-8	40	*his3-1, leu2-0 ,ura3-0, ypr144c::kanMX4 ,pNOPPA1-noc4-8 (LEU2)*	Milkereit et al., 2003
BY4742	207	*his3-1 leu2-0 lys2-0 ura3-0*	Euroscarf
BY4742 NOC4-TAP	96	*his3-1, leu2-0, lys2-0, ura3-0, noc4::NOC4-TAP-URA3*	This study, derivate of BY4742
BY4742 UTP4-TAP	1907	*his3-1, leu2-0, lys2-0, ura3-0, utp4::UTP4-TAP-URA3*	This study, derivate of BY4742
pGAL-RPS5	323	*his3-1, leu2-0, ura3-0, yjr123w::kanMX4, YCplac111-pGAL-RPS5 (LEU2)*	Ferreira-Cerca et al., 2005
pGAL-RPS5 NOC4-TAP	1241	*his3-1, leu2-0, ura3-0, yjr123w::kanMX4, noc4::NOC4-TAP-URA3, YCplac111-pGAL-RPS5 (LEU2)*	This study, derivate of TY323
pGAL-RPS5 PWP2-TAP	1525	*his3-1, leu2-0, ura3-0, yjr123w::kanMX4, pwp2::PWP2-TAP-URA3, YCplac111-pGAL-RPS5 (LEU2)*	This study, derivate of TY323
pGAL-RPS5 UTP4-TAP	1524	*his3-1, leu2-0, ura3-0, yjr123w::kanMX4, utp4::UTP4-TAP-URA3, YCplac111-pGAL-RPS5 (LEU2)*	This study, derivate of TY323
pGAL-RPS9	259	*his3-1, leu2-0, ura3-0, ybr189w::kanMX4, ypl081w::HIS3, YCplac111-pGAL-RPS9A (LEU2)*	Ferreira-Cerca et al., 2005
pGAL-RPS9 NOC4-TAP	1887	*his3-1, leu2-0, ura3-0, ybr189w::kanMX4, ypl081w::HIS3, noc4::NOC4-TAP-URA3, YCplac111-pGAL-RPS9A (LEU2)*	This study, derivate of TY259
pGAL-RPS9 PWP2-TAP	1884	*his3-1, leu2-0, ura3-0, ybr189w::kanMX4, ypl081w::HIS3, pwp2::PWP2-TAP-URA3, YCplac111-pGAL-RPS9A (LEU2)*	This study, derivate of TY259
pGAL-RPS9 UTP4-TAP	1883	*his3-1, leu2-0, ura3-0, ybr189w::kanMX4, ypl081w::HIS3, utp4::UTP4-TAP-URA3, YCplac111-pGAL-RPS9A (LEU2)*	This study, derivate of TY259
pGAL-RPS11A	325	*his3-1 leu2-0 ura3-0 ybr048w::kanMX4 ydr025w::HIS3 YCplac111-pGAL-RPS11A (LEU2)*	Ferreira-Cerca et al., 2005
pGAL-RPS11A NOC4-TAP	1892	*his3-1, leu2-0, ura3-0, ybr048w::kanMX4, ydr025w::HIS3, noc4::NOC4-TAP-URA3, YCplac111-pGAL-RPS11A (LEU2)*	This study, derivate of TY325
pGAL-RPS11A PWP2-TAP	1889	*his3-1, leu2-0, ura3-0, ybr048w::kanMX4, ydr025w::HIS3, pwp2::PWP2-TAP-URA3, YCplac111-pGAL-RPS11A (LEU2)*	This study, derivate of TY325
pGAL-RPS11A UTP4-TAP	1888	*his3-1, leu2-0, ura3-0, ybr048w::kanMX4, ydr025w::HIS3, utp4::UTP4-TAP-URA3, YCplac111-pGAL-RPS11A (LEU2)*	This study, derivate of TY325
pGAL-RPS13	701	*his3-1, leu2-0, ura3-0, ydr064w::kanMX4, YCplac111-pGAL-RPS13 (LEU2)*	Ferreira-Cerca et al., 2005
pGAL-RPS13 NOC4-TAP	1897	*his3-1, leu2-0, ura3-0, ydr064w::kanMX4, noc4::NOC4-TAP-URA3, YCplac111-pGAL-RPS13 (LEU2)*	This study, derivate of TY701
pGAL-RPS13 PWP2-TAP	1894	*his3-1, leu2-0, ura3-0, ydr064w::kanMX4, pwp2::PWP2-TAP-URA3, YCplac111-pGAL-RPS13 (LEU2)*	This study, derivate of TY701
pGAL-RPS13 UTP4-TAP	1893	*his3-1, leu2-0, ura3-0, ydr064w::kanMX4, utp4::UTP4-TAP-URA3, YCplac111-pGAL-RPS13 (LEU2)*	This study, derivate of TY701
pGAL-RPS14	399	*his3-1, leu2-0, ura3-0, ycr031c::HIS3MX6, yjl191w::kanMX4,*	Ferreira-Cerca et al.,

		Ycplac111-pGAL-RPS14 (LEU2)	2005
pGAL-RPS14 NOC4-TAP	2108	*his3-1, leu2-0, ura3-0, ycr031c::HIS3MX6, yjl191w::kanMX4, noc4::NOC4-TAP-URA3, Ycplac111-pGAL-RPS14 (LEU2)*	This study, derivate of TY399
pGAL-RPS14 PWP2-TAP	2105	*his3-1, leu2-0, ura3-0, ycr031c::HIS3MX6, yjl191w::kanMX4, pwp2::PWP2-TAP-URA3, Ycplac111-pGAL-RPS14 (LEU2)*	This study, derivate of TY399
pGAL-RPS14 UTP4-TAP	2104	*his3-1, leu2-0, ura3-0, ycr031c::HIS3MX6, yjl191w::kanMX4, utp4::UTP4-TAP-URA3, Ycplac111-pGAL-RPS14 (LEU2)*	This study, derivate of TY399
pGAL-RPS15	89	*his3-1, leu2-0, ura3-0, yol040c::kanMX4, pFL36-pGalRPS15 (LEU2)*	Léger-Silvestre et al., 2004
pGAL-RPS15 NOC4-TAP	90	*his3-1, leu2-0, ura3-0, yol040c::kanMX4, noc4::NOC4-TAP-URA3, pFL36-pGalRPS15 (LEU2)*	Léger-Silvestre et al., 2004
pGAL-RPS15 PWP2-TAP	1529	*his3-1, leu2-0, ura3-0, yol040c::kanMX4, pwp2::PWP2-TAP-URA3, pFL36-pGalRPS15 (LEU2)*	This study, derivate of TY89
pGAL-RPS15 UTP4-TAP	1528	*his3-1, leu2-0, ura3-0, yol040c::kanMX4, utp4::UTP4-TAP-URA3, pFL36-pGalRPS15 (LEU2)*	This study, derivate of TY89
RPS22A-Shuffle	Y1425	*his3-1, leu2-0, ura3-0, yjl190c::kanMX4, ylr367w::HIS3MX6, YCplac33-RPS22A (URA3)*	This study, yeast strains Y1429 and Y1239 were crossed, and the resulting geneticin resistant and histidin prototroph diploid wastransformed with vector K977. Tetrade analysis was performed and a 5-fluorooritic acid sensitive, histidin prototroph and geneticin resistant spore was selected
dRPS22A	Y1429	*his3-1, leu2-0, ura3-0, yjl190c::kanMX4*	This study, tetrade analysis of Euroscarf strain Y31235 was performed and a geneticin resistant spore was selected
dRPS22B (HIS3MX6)	Y1239	*his3-1, leu2-0, ura3-0, met15-0, ylr367w::HIS3MX6*	This study, the kanMX4 cassette in Euroscarf strain Y05276 was exchanged by a HIS3MX6 cassette by transformation with a BglI/PmeI digest of vector pFA6a-GFP(S65T) (Longtine et al., 1998)
pGal-RPS22A	1427	*his3-1, leu2-0, ura3-0, yjl190c::kanMX4, ylr367w::HIS3MX6, YCplac111-pGAL-RPS22A (LEU2)*	This study, yeast strain Y1425 was transformed with vector K750. 5-Fluorooritic acid resistant clones were selected which grew on medium containing galactose as carbon source and stopped growing on glucose containing medium
pGAL-RPS22	1902	*his3-1, leu2-0, ura3-0, yjl190c::kanMX4, ylr367w::HIS3MX6,*	This study, derivate of

NOC4-TAP		*noc4::NOC4-TAP-URA3, YCplac111-pGAL-RPS22A (LEU2)*	TY1427
pGAL-RPS22 PWP2-TAP	1899	*his3-1, leu2-0, ura3-0, yjl190c::kanMX4, ylr367w::HIS3MX6, pwp2::PWP2-TAP-URA3, YCplac111-pGAL-RPS22A (LEU2)*	This study, derivate of TY1427
pGAL-RPS22 UTP4-TAP	1898	*his3-1, leu2-0, ura3-0, yjl190c::kanMX4, ylr367w::HIS3MX6, utp4::UTP4-TAP-URA3, YCplac111-pGAL-RPS22A (LEU2)*	This study, derivate of TY1427
pGAL-FLAG-NOC4	405	*his3-1, leu2-0, ura3-0, ypr144c::kanMX4, YCplac111-pGAL-FLAG-NOC4 (LEU2)*	This study, derivate of TY30/NOC4-Shuffle (Kühn et al. 2009)
pGAL-NOC4 UTP4-TAP	1903	*his3-1, leu2-0, ura3-0, ypr144c::kanMX4, utp4::UTP4-TAP-URA3, YCplac111-pGAL-FLAG-NOC4 (LEU2)*	This study, derivate of TY405
pGAL-NOC4 PWP2-TAP	1904	*his3-1, leu2-0, ura3-0, ypr144c::kanMX4, pwp2::PWP2-TAP-URA3, YCplac111-pGAL-FLAG-NOC4 (LEU2)*	This study, derivate of TY405
pGAL-NOC4 UTP22-TAP	1905	*his3-1, leu2-0, ura3-0, ypr144c::kanMX4, utp22::UTP22-TAP-URA3, YCplac111-pGAL-FLAG-NOC4 (LEU2)*	This study, derivate of TY405
pGAL-NOC4 IMP3-TAP	1906	*his3-1, leu2-0, ura3-0, ypr144c::kanMX4, imp3::IMP3-TAP-URA3, YCplac111-pGAL-FLAG-NOC4 (LEU2)*	This study, derivate of TY405
pGAL-NOC4 ENP1-TAP	2112	*his3-1, leu2-0, ura3-0, ypr144c::kanMX4, enp1::ENP1-TAP-URA3, YCplac111-pGAL-FLAG-NOC4 (LEU2)*	This study, derivate of TY405
