# Supplementary material for: Interrelationships between Yeast Ribosomal Protein Assembly Events and Transient Ribosome Biogenesis Factors Interactions in Early Pre-Ribosomes
Source: PLoS One. 2012 Mar 14;7(3):e32552. doi: 10.1371/journal.pone.0032552 (PMC3303783; doi:10.1371/journal.pone.0032552)
Supplement: Figure S3 — Oligos used in this study. (PDF) [file pone.0032552.s003.pdf]

Fig. S3 Oligonucleotides used in this study

**Name**	**O**ligo	**Sequence**
Noc4- TAP-F	190	AGCGAAGCGTCATCCCAAGGTAACGTCTACTTGCCTGGGGTAGCATGGTCCATGGAAAAGAGAAG
Noc4- TAP-R	189	TAAATGTATATATACACACTTTTAAAACTTCGTTGTTCATCTTCGCTTTACGACTCACTATAGGG
Pwp2-TAP-F	1809	AATGAAAATGATTCCAGTGATGAAGAAGAAAATGAGAAAGAGCTTCCTTCCATGGAAAAGAGAAG
Pwp2-TAP-R	1810	GGGAATACATGATGTGAATGTGCATAAATAGAGGACAGTGAATATTTTTACGACTCACTATAGGG
Utp4-TAP-F	1813	ACTTTTCACTCCAAACAAAAGGCGTTTATTCAACCAAAGTTAGTGTTTTCCATGGAAAAGAGAAG
Utp4-TAP-R	1814	GCCTTTTAATAGCATCTCTCTATTCTTCGGTATGTTGACTTAAATTAATACGACTCACTATAGGG
Utp22-TAP-F	2261	GAGATTGCTGCATTCGGGAATGACATGGTTATAAATTTTGAGACAGATTCCATGGAAAAGAGAAG
Utp22-TAP-R	2262	TTTAATATTATACAGATACTTCTAAAAGTTATGATTTTGTTGTTTATTTACGACTCACTATAGGG
Imp3-TAP-F	2267	AAAACCTTGTTGAGATACAGAAACCAAATCGACGATTTTGATTTTTCATCCATGGAAAAGAGAAG
Imp3-TAP-R	2268	AGATGATAAATCGAGTATTGATACAGAAAACGTAAGTTTGTAGTCAATTACGACTCACTATAGGG
ENP1-pBS1479-INT-UP	2318	TTTGTTGATCCACAGGAAGCTAATGATGATTTAATGATTGATGTCAATTCCATGGAAAAGAGAAG
ENP1-pBS1479-INT-DO	2319	GGGAAAGACCGAGCGATATAAAATTGATGAAAAATTGATATTACAGCATACGACTCACTATAGGG
18S probe	205	CATGGCTTAATCTTTGAGAC
D-A_2 probe	1819	GTAAAAGCTCTCATGCTCTTGCC
RPS 22A F	398	GGCGGCGAGCTCGTTAAATTGGTACTGTGC
RPS 22A R	399	GCCGCCGGTACC TTTACTCTATTAACGCAT
RP22A_GAL_F	473	CGCCGCGGATCCATGACCAGATCTTCCGTT
S22A_R_PstI	1105	CGCCGCCTG CAGTTTACTCTATTAACGCAT
