# Supplementary material for: Interrelationships between Yeast Ribosomal Protein Assembly Events and Transient Ribosome Biogenesis Factors Interactions in Early Pre-Ribosomes
Source: PLoS One. 2012 Mar 14;7(3):e32552. doi: 10.1371/journal.pone.0032552 (PMC3303783; doi:10.1371/journal.pone.0032552)
Supplement: Figure S4 — Plasmids used in this study. (PDF) [file pone.0032552.s004.pdf]

Fig. S4 Plasmids used in this study

Name	number	Features	Origin
pRPS28-FLAG-RPS3	424	pRPS28-FLAG-RPS3 2µ URA3	Ferreira-Cerca et al., 2007
pRPS28-FLAG-RPS5	351	pRPS28-FLAG-RPS5 2µ URA3	Ferreira-Cerca et al., 2007
pRPS28-RPS9A-FLAG	999	pRPS28-RPS9A-FLAG 2µ URA3	Ferreira-Cerca et al., 2007
pRPS28-FLAG-RPS11A	1001	pRPS28-FLAG-RPS11A 2µ URA3	Ferreira-Cerca et al., 2007
pRPS28-FLAG-RPS13	427	pRPS28-FLAG-RPS13 ORI 2µ URA3	Ferreira-Cerca et al., 2007
pRPS28-FLAG-RPS14A	1111	R pRPS28-FLAG-PS14A 2µ URA3	Ferreira-Cerca et al., 2007
pRPS28-FLAG-RPS15	429	pRPS28-FLAG-RPS15 2µ URA3	Ferreira-Cerca et al., 2007
pRPS28-FLAG-RPS16A	622	pRPS28-FLAG-RPS16A 2µ URA3	Ferreira-Cerca et al., 2007
pRPS28-FLAG-RPS19A	432	pRPS28-FLAG-RPS19A 2µ URA3	Ferreira-Cerca et al., 2007
pRPS28-FLAG-RPS20	623	pRPS28-FLAG-RPS20 2µ URA3	Ferreira-Cerca et al., 2007
YCplac111-pGAL	230	pGAL ARS1 CEN4 LEU2	Ferreira-Cerca et al., 2005
Ycplac33-RPS22A	977	RPS22A ARS1 CEN4 URA3	PCR product obtained with oligos O398 and O399 and yeast genomic DNA was cloned SacI / KpnI into vector Ycplac33
YCplac111-pGAL-RPS22A	750	pGAL-RPS22A ARS1 CEN4 LEU2	PCR product obtained with oligos O473 and O1105 and yeast genomic DNA was cloned BamHI / PstI into vector K230, correct sequence of the insert was confirmed
